# LncRNA *SOX9-AS1* triggers a transcriptional program involved in lipid metabolic reprogramming, cell migration and invasion in triple-negative breast cancer

**DOI:** 10.1038/s41598-024-51947-2

**Published:** 2024-01-17

**Authors:** Mireya Cisneros-Villanueva, Marco Antonio Fonseca-Montaño, Magdalena Ríos-Romero, César López-Camarillo, Silvia Jiménez-Morales, Elizabeth Langley, Alan Sajid Rosette-Rueda, Alberto Cedro-Tanda, Daniel Hernández-Sotelo, Alfredo Hidalgo-Miranda

**Affiliations:** 1 Laboratorio Genómica del Cáncer, Instituto Nacional de Medicina Genómica (INMEGEN), 14610 Mexico, México; 2 Programa de Doctorado en Ciencias Biomédicas, Facultad de Ciencias Químico Biológicas, Universidad Autónoma de Guerrero (UAGro), Chilpancingo de los Bravo, Guerrero México; 3 Laboratorio de Epigenética del Cáncer, Facultad de Ciencias Químico Biológicas, Universidad Autónoma de Guerrero (UAGro), Chilpancingo de los Bravo, Guerrero México; 4 Programa de Doctorado, Posgrado en Ciencias Biológicas, Unidad de Posgrado, Universidad Nacional Autónoma de México (UNAM), 04510 Mexico, México; 5 Posgrado en Ciencias Genómicas, Universidad Autónoma de la Ciudad de México, Mexico, México; 6 Laboratorio de Cáncer Hormono Regulado, Instituto Nacional de Cancerología (INCan), 14080 Mexico, México; 7 Instituto Nacional de Medicina Genómica (INMEGEN), 14610 Mexico, México

**Keywords:** Oncology, Long non-coding RNAs, Cell migration, Cell invasion, Epithelial-mesenchymal transition, Gene expression analysis, Lipids, Fatty acids, Cancer, Breast cancer, Cancer genomics, Cancer metabolism

## Abstract

At the molecular level, triple-negative breast cancer (TNBC) is frequently categorized as PAM50 basal-like subtype, but despite the advances in molecular analyses, the clinical outcome for these subtypes is uncertain. Long non-coding RNAs (lncRNAs) are master regulators of genes involved in hallmarks of cancer, which makes them suitable biomarkers for breast cancer (BRCA) diagnosis and prognosis. Here, we evaluated the regulatory role of lncRNA *SOX9-AS1* in these subtypes*.* Using the BRCA-TCGA cohort, we observed that *SOX9-AS1* was significantly overexpressed in basal-like and TNBC in comparison with other BRCA subtypes. Survival analyzes showed that *SOX9-AS1* overexpression was associated with a favorable prognosis in TNBC and basal-like patients. To study the functions of *SOX9-AS1*, we determined the expression levels in a panel of nine BRCA cell lines finding increased levels in MDA-MB-468 and HCC1187 TNBC. Using subcellular fractionation in these cell lines, we ascertained that *SOX9-AS1* was located in the cytoplasmic compartment. In addition, we performed *SOX9-AS1* gene silencing using two short-harping constructs, which were transfected in both cell models and performed a genome-wide RNA-seq analysis. Data showed that 351 lncRNAs and 740 mRNAs were differentially expressed in MDA-MB-468 while 56 lncRNAs and 100 mRNAs were modulated in HCC1187 cells (Log2FC < - 1.5 and > 1.5, p.adj value < 0.05). Pathway analysis revealed that the protein-encoding genes potentially regulate lipid metabolic reprogramming, and epithelial–mesenchymal transition (EMT). Expression of lipid metabolic-related genes *LIPE*, *REEP6*, *GABRE*, *FBP1*, *SCD1*, *UGT2B11*, *APOC1* was confirmed by RT-qPCR. Functional analysis demonstrated that the knockdown of *SOX9-AS1* increases the triglyceride synthesis, cell migration and invasion in both two TNBC cell lines. In conclusion, high *SOX9-AS1* expression predicts an improved clinical course in patients, while the loss of *SOX9-AS1* expression enhances the aggressiveness of TNBC cells.

## Introduction

Triple-negative breast cancer (TNBC) is a heterogeneous BRCA subtype characterized by the lack of estrogen receptor (ER), progesterone receptor (PR), and human epidermal growth factor receptor 2 (HER2) expression, as determined by immunohistochemical staining^1^. At the molecular level, subtyping using the PAM50 gene signature often classifies BRCA into four intrinsic subtypes: luminal A, luminal B, HER2-enriched and basal-like^2–6^. As high as 60–80% of basal-like tumors are categorized as TNBC, nevertheless, the remaining basal-like tumors (expressing receptors) are grouped into other molecular subtypes^7,8^. The heterogeneity of TNBC and lack of molecular targets results in a diversity of treatment approaches unguided by tumor biology^9^. Therefore, a deeper characterization of the hallmarks of TNBC is necessary for identifying the events that drive oncogenesis. A pathogenic outcome can be accomplished by affecting metabolic reprogramming and other processes in TNBC cells^10^. However, more exhaustive oncogenomic analyses of TNBCs are required to understand the heterogeneity of the disease^11^.

LncRNAs are non-protein coding transcripts involved in diverse biological phenomena, and their specific patterns of expression coordinate different normal and disease processes^12,13^. In cancer, lncRNAs play a significant role in the regulation of gene expression and are involved in multiple hallmarks, suggesting that these molecules might be used as diagnostic or prognostic biomarkers^14,15^. However, given the vast number of lncRNAs identified using transcriptomic studies, it is critical to develop effective strategies for studying their roles and mechanisms in cancer^16,17^. In this regard, our research group previously evaluated lncRNA expression profiles in 156 TNBC biopsies through microarray expression. In this study, 710 lncRNAs showed differential expression between TNBC molecular subtypes, and most of these lncRNAs have not been characterized. One of these deregulated lncRNAs was *SOX9-AS1*^18^. In another study including 74 tumors classified by PAM50, we found that *SOX9-AS1* was significantly overexpressed in basal-like tumors^19^. However, the biological role of *SOX9-AS1* in this BRCA subtype is poorly understood.

*SOX9-AS1* is a human lncRNA, transcribed from cytogenetic band 17q24.3 and measuring 3033 bp^20^. It has been established that *SOX9-AS1* promotes hepatocellular carcinoma (HCC) progression*,* through its sponge function of *miR-5590-3p,* causing *SOX9* to be overexpressed. In turn, *SOX9* activates *SOX9-AS1* expression^21^. A meta-analysis identified *SOX9-AS1* as part of a signature deregulated lncRNA-miRNA-mRNA network for TNBC. Taken together, this evidence suggests that *SOX9-AS1* might have an important biological and even prognostic role in basal-like and TNBC. However, there is a need for comprehensive examination of this lncRNA expression profile to characterize its expression, molecular functions, and roles in TNBC^22^.

In this study, we analyzed *SOX9-AS1* expression in a BRCA-TCGA cohort and determined its prognostic value in TNBC and the PAM50 basal-like subtype. Additionally, we elucidate its biological role through differential gene expression (DGE) analysis, KEGG pathways, Gene Ontology and GSEA in these subtypes from the same cohort. Moreover, transcriptome analysis using RNA-Seq of *SOX9-AS1* deficient-cells indicate that silencing of this lncRNA regulates a plethora of protein-encoding genes that increases progression of two different TNBC cell lines. Therefore, we propose an onco-suppressor biological and clinical role for this novel lncRNA in TNBC and basal-like BRCA subtypes.

## Results

### SOX9-AS1 exhibits different expression patterns in multiple tissue types

**Figure 1:**
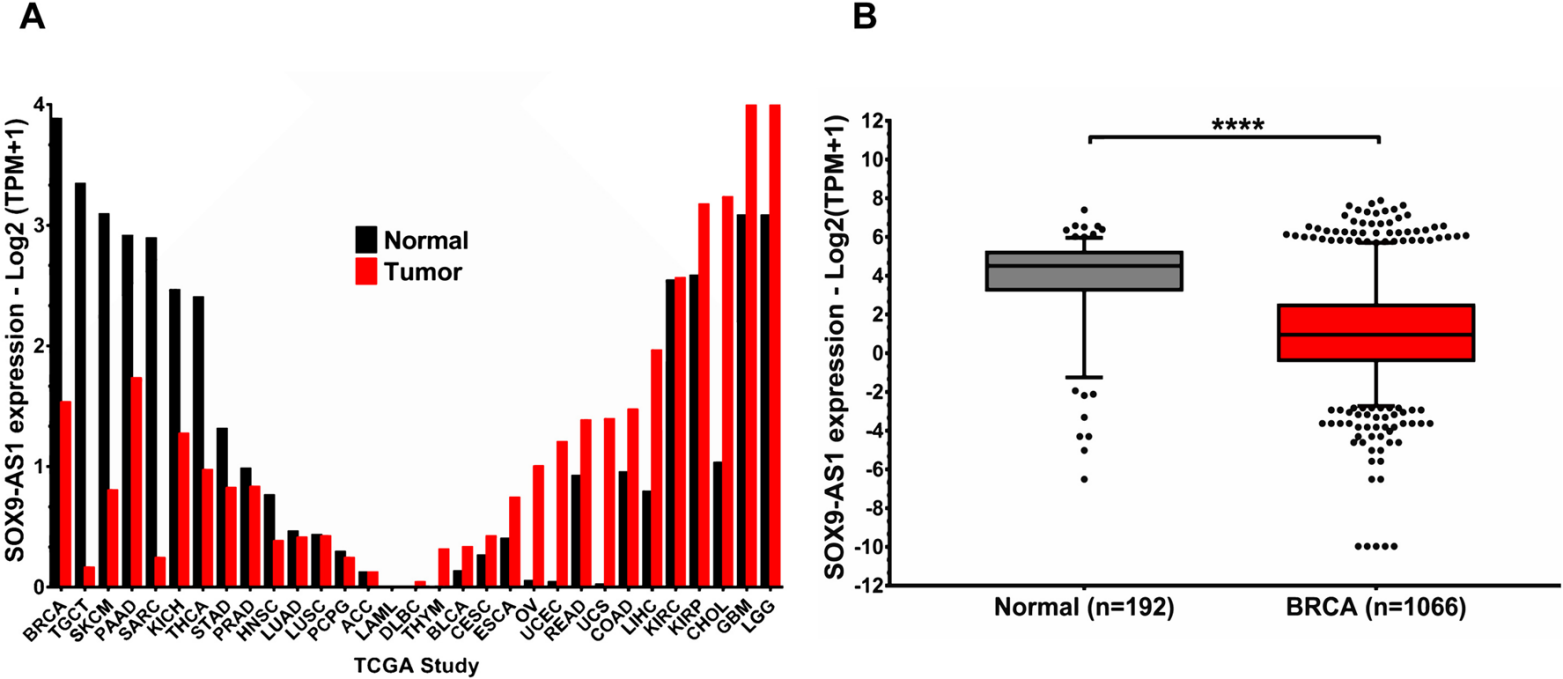
*SOX9-AS1* expression is deregulated in different human tumors. (**A**) *SOX9-AS1* expression profile across all tumor samples and normal tissues (bar plot). The height of bar represents the median expression of tumor (red bars) and normal tissue (black bars) (data obtained from GEPIA2 platform). (**B)** *SOX9-AS1* expression in normal breast tissue samples *versus* BRCA tissue determined by RNA-seq from the BRCA-TCGA cohort. Statistical differences between groups were compared using the Mann–Whitney test, ****p < 0.0001.

Few studies have reported *SOX9-AS1* expression levels in tumors^21,23^. In this regard, we determined its expression pattern across 31 cancer types using the TCGA database and the GEPIA2 platform. We found that *SOX9-AS1* was overexpressed in 13 different normal tissues *versus* samples from diverse tumors (BRCA, TGCT, SKCM, PAAD, SARC, KICH, THCA, STAD, PRAD, HNSC, LUAD, LUSC, and PCPG), while it was underexpressed in 17 different normal tissues *versus* tumors (DLBC, THYM, BLCA, CESC, ESCA, OV, UCEC, READ, UCS, COAD, LIHC, KIRC, KIRP, CHOL, GBM, and LGG). Additionally, we found one tumor type with the same expression level (ACC), and one non-solid tumor without expression (LAML) (Fig. 1A). As shown in Fig. 1B, *SOX9-AS1* expression was significantly downregulated in BRCA samples (p value = 0.0001) compared to normal tissues. These results suggest that *SOX9-AS1* could have a relevant role in cancer biology.

### High SOX9-AS1 expression in TNBC and basal-like is associated with better prognosis

**Figure 2:**
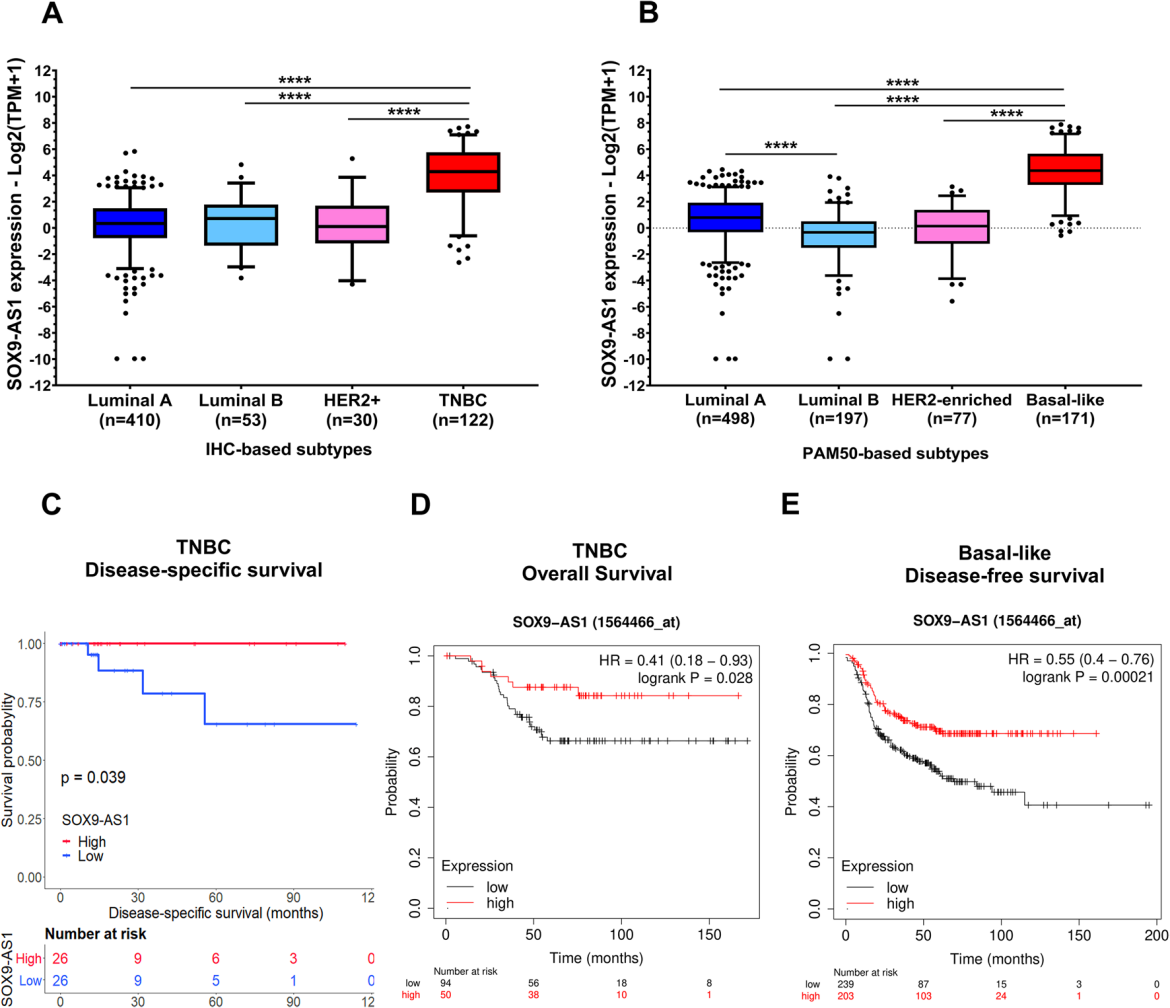
High *SOX9-AS1* expression is associated with better prognosis in basal-like and TNBC. *SOX9-AS1* expression between (**A**) receptor status (IHC), and (**B**) molecular subtypes classified by PAM50. The horizontal lines in the box plots represent the medians, the boxes represent the interquartile ranges, and the whiskers represent the 5th and 95th percentiles. RNA-seq data from the BRCA-TCGA cohort. Dunn's multiple comparisons and Kruskal–Wallis tests, ****p < 0.0001. (**C**) Kaplan–Meier curve of TNBC patients classified into high- and low-risk groups using the upper and lower quartiles of *SOX9-AS1* expression for disease-specific survival (the analysis was conducted on 144 patients). Log-rank test between patients with low fold-change ( < -1.5) and high fold-change (> 1.5) and p value < 0.05. (**D**) Overall Survival in TNBC patients (the analysis was conducted on 144 patients), and (**E**) disease-free survival in basal-like patients, using Kaplan–Meier curves (the analysis was conducted on 442 patients), determined by Kaplan–Meier Plotter (breast). In red: patients with expression of *SOX9-AS1* above the automatic cutoff calculation. In black, patients with expressions of *SOX9-AS1* below automatic cutoff calculation.

Next, we determined the expression level of *SOX9-AS1* among tumor subtypes classified by IHC and the molecular PAM50 classifier. Notably, the expression of *SOX9-AS1* was higher in the TNBC and basal-like molecular subtypes compared to receptor-positive BRCA (p value = 0.0001) (Fig. 2A,B). As shown in Supplementary Table 1, there was a significant association between *SOX9-AS1* expression and ER (p value = 5.53e−21) and PR (p value = 4.94e−45) status, being higher in hormone receptor-negative samples. Furthermore, when using PAM50 to discriminate molecular subtypes (p value = 5.449e−50) there was a clear enrichment of *SOX9-AS1* in basal-like tissue. Our results suggest that the high expression of *SOX9-AS1* is a possible biomarker for TNBC and basal-like subtypes.

To investigate the clinical value of *SOX9-AS1* expression in TNBC and basal-like patients, Kaplan–Meier survival analyses were performed. As shown in Fig. 2C, TCGA TNBC patients have an increased disease-specific survival (DSS) time in the high *SOX9-AS1* expression group, compared with those expressing low levels (Log-rank test, p value = 0.039). To discern if the prognostic value of *SOX9-AS1* was replicated in independent cohorts, we used the Kaplan–Meier plotter service to analyze the *SOX9-AS1* expression data^24,25^. We found that high expression of *SOX9-AS1* was associated with an increased overall survival (OS) in TNBC patients (HR = 0.41, 95% CI (0.18–0.93); Log-rank p value = 0.028) (Fig. 2D). Overexpression of *SOX9-AS1* was also related to a significant increase in disease-free survival (DFS) in basal-like patients (HR = 0.55, 95% CI (0.4–0.76); Log-rank test, p value = 0.00021) (Fig. 2E). These data indicate that high *SOX9-AS1* expression predicts an increased DSS, OS, and DFS in TNBC and basal-like patients.

### SOX9-AS1 potentially regulates processes involved in metabolism in TNBC and Basal-like samples from TCGA cohort

**Figure 3:**
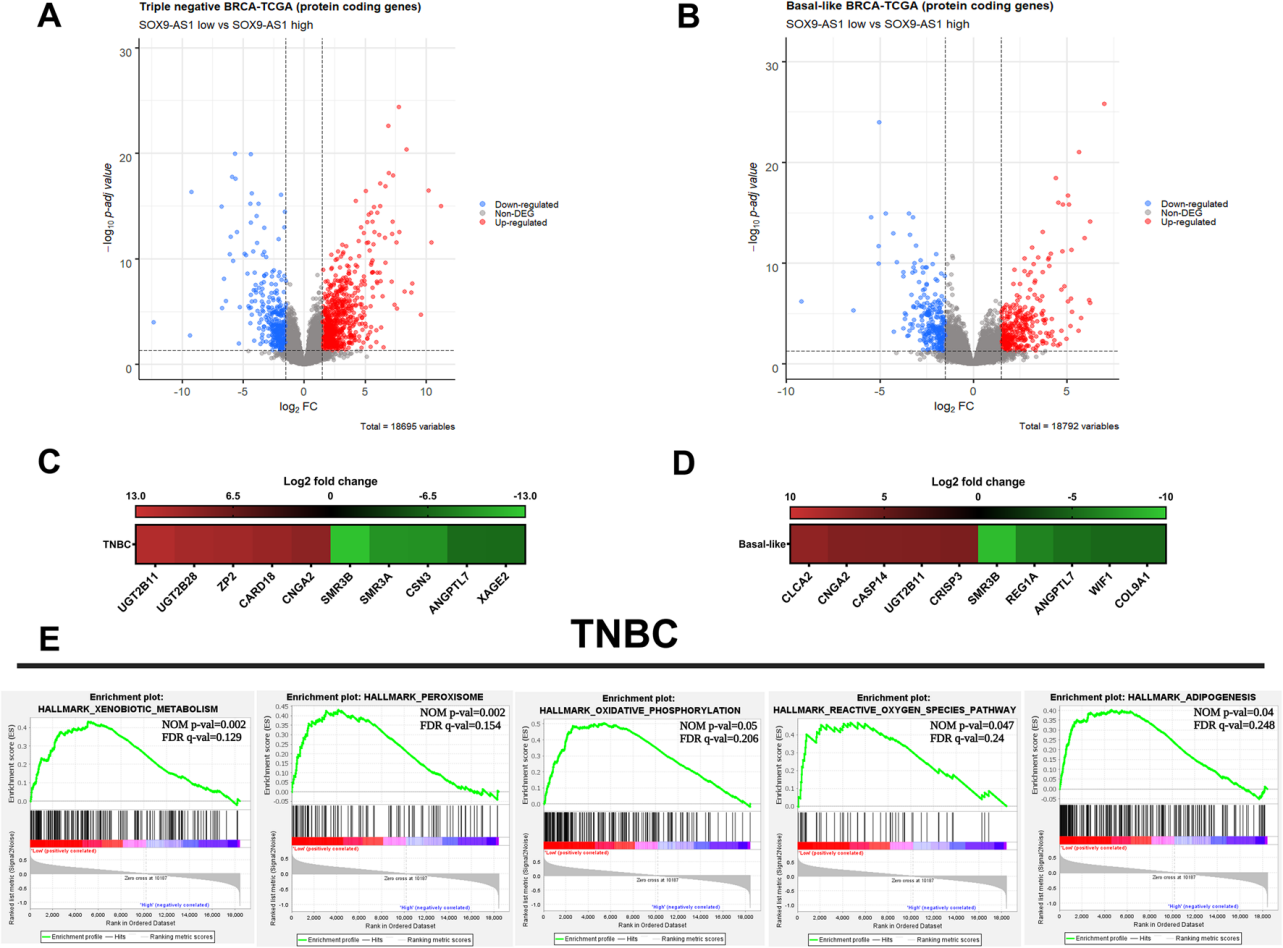
GSEA analyses of DEGs in TNBC and basal-like samples of the BRCA-TCGA cohort. The Volcano plots represent the distribution of DEGs. Blue genes are downregulated, red genes are upregulated, and gray genes are not differentially expressed in (**A**) TNBC-TCGA, and (**B**) basal-like-TCGA samples. Up- and downregulated genes were defined as Log2FC < - 1.5 and > 1.5 with adjusted p value < 0.05. Heatmap of the DGEs contributing to top 5 in (**C**) TNBC and (**D**) basal-like samples. Green: low expression, red: high expression according to Log2 fold change (visualized on the color bar above the heatmap). (**E**) Using the Hallmark gene set, genes involved in xenobiotic metabolism, peroxisome pathway, oxidative phosphorylation, reactive oxygen species pathway, and adipogenesis were significantly enriched in TNBC samples with FDR q value < 0.25. The NOM p value and FDR q value are displayed.

To clarify the role of *SOX9-AS1* in gene regulation and biology of basal-like and TNBC, we performed a DGE analysis between the *SOX9-AS1* high expression group and the *SOX9-AS1* low expression group (see “Materials and methods” for details). We identified 1,173 differentially expressed genes (DEGs) in TNBC, of which 791 (67.4%) were overexpressed and 382 (32.6%) were underexpressed (Fig. 3A). In basal-like samples we identified 619 DEGs of which 352 (56.9%) were overexpressed and 267 (43.1%) underexpressed (Fig. 3B) (Supplementary Table 2). Figure 3C,D shows the top 5 DEGs in TNBC and basal-like, respectively. 428 genes were shared between TNBC and basal-like subtypes (Supplementary Fig. 1A; Supplementary Table 3). In addition, we identified 697 differentially expressed lncRNAs of which 481 (69%) lncRNAs were overexpressed and 216 (31%) were underexpressed in TNBC (Supplementary Fig. 1B,C). In contrast, the basal-like subtype only presented 343 differentially expressed lncRNAs, 189 (55.1%) lncRNAs overexpressed and 154 (44.9%) lncRNAs underexpressed (Supplementary Fig. 1D,E; Supplementary Table 4).

To elucidate the cellular processes and pathways of DEGs regulated by *SOX9-AS1* in TNBC and basal-like subtypes, we performed GO and KEGG pathway analyses. We observed that similar processes and functions were shared across both TNBC and basal-like subtypes (Supplementary Fig. 2A,B). GO analysis showed numerous genes associated with regulation of hydrolase activity, skin development and differentiation, extracellular matrix structural organization, and hormone/xenobiotic metabolic process were significatively enriched (Supplementary Table 5). KEGG pathway enrichment analysis revealed that DEGs were significantly enriched in neuroactive ligand-receptor interaction, calcium signaling pathway, metabolism of organic compounds, protein absorption and digestion, and secretion of saliva and gastric acid (Supplementary Fig. 2C,D; Supplementary Table 6). Altogether, these results indicate that *SOX9-AS1* could regulate a plethora of genes mostly involved in the metabolism of organic and chemical compounds in TNBC and basal-like BRCA samples.

In order to reduce variation and redundancy and to provide a narrower view of the biological role of *SOX9-AS1*, we performed a GSEA analysis using the Hallmark gene set^26^ with the same DEGs in TNBC and basal-like samples from the BRCA-TCGA cohort. The results with a NOM (p value < 0.05) and FDR (q value < 0.25) showed expression patterns associated with xenobiotic metabolism (i.e., *BLVRB, CYP4F2, TMBIM6*), peroxisome pathway (i.e., *SULT2B1, DHCR24, IDH2*), oxidative phosphorylation (i.e., *PDHB, UQCRQ, POR*), reactive oxygen species pathway (i.e., *HMOX2, G6PD, PTPA*), and adipogenesis (i.e., *REEP6, CMBL, TKT*) in TNBC samples (Fig. 3E). However, in basal-like samples, we did not identify significantly enriched Hallmark gene sets, although the same Hallmarks gene sets reported in TNBC were observed (Supplementary Table 7). Collectively, this suggests that *SOX9-AS1* might regulate genes directly related to metabolic pathways in TNBC samples.

### Relationship between SOX9-AS1 expression and protein-coding genes of TNBC and Basal-like samples

**Figure 4:**
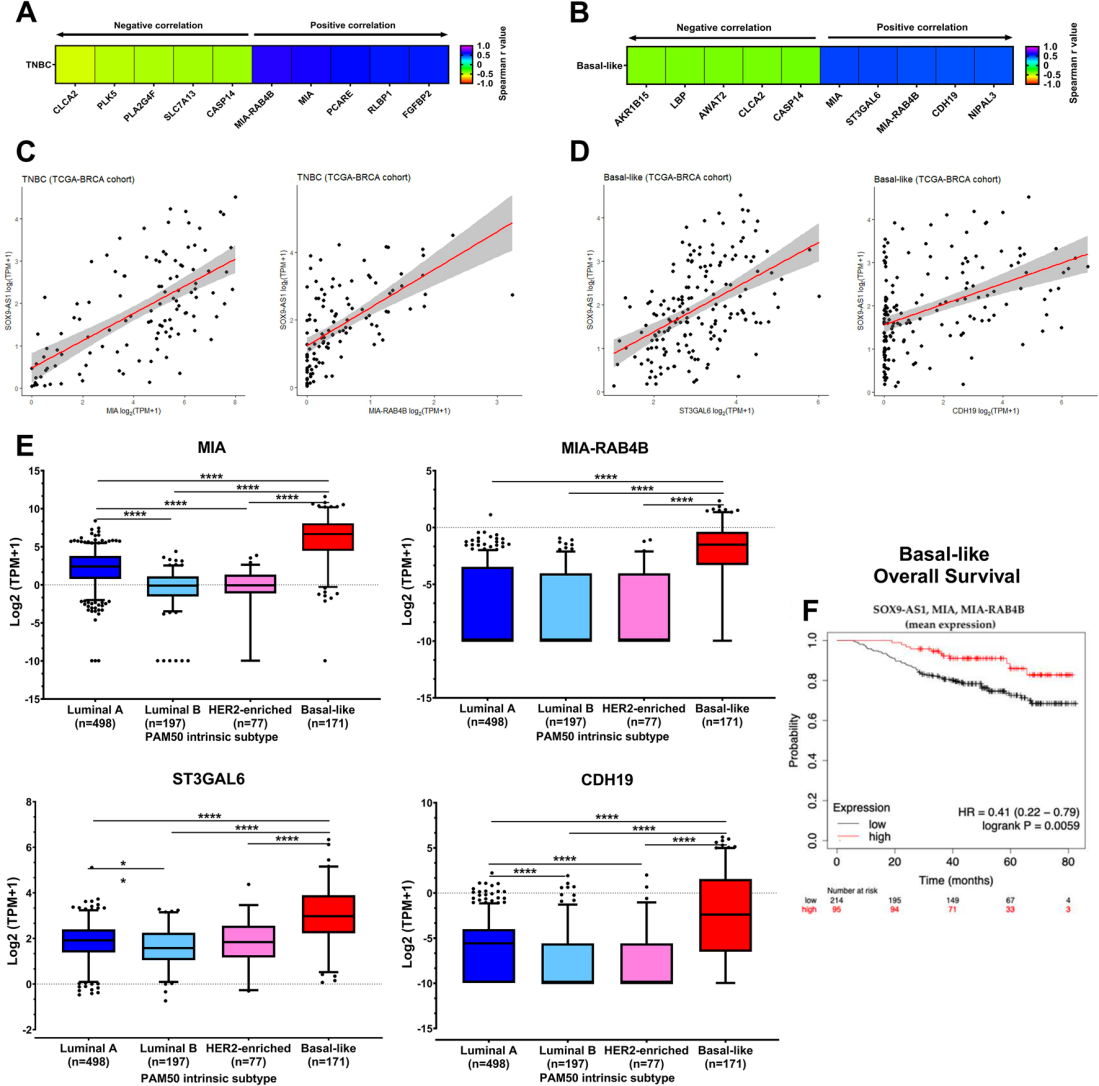
Correlation of *SOX9-AS1* expression with genes in TNBC and basal-like samples from BRCA-TCGA cohort. Heatmap showing the top 5 genes that show the greatest negative and positive correlation with the *SOX9-AS1* expression in (**A**) TNBC and (**B**) basal-like samples. (**C**) Correlation plots of *SOX9-AS1* expression with *MIA*, and *MIA-RAB4B* in TNBC. (**D**) Correlation plots of *SOX9-AS1* expression with *ST3GAL6*, *CDH19* in basal-like samples. Spearman's rank correlation critical value was used for the analysis. (**E**) Expression level of *MIA*, *MIA-RAB4B, ST3GAL6*, and *CDH19* between molecular subtypes classified by PAM50 (determined by RNA-seq in the BRCA-TCGA cohort). Dunn's multiple comparisons and Kruskal–Wallis tests (****p < 0.0001). (**F**) Kaplan–Meier curve depicting the influence of *SOX9-AS1, MIA* and *MIA-RAB4B* mean expression on Overall Survival probability for basal-like patients (the analysis was conducted on 309 patients)*.*

Given that predicting functions of lncRNAs remains a substantial challenge^27^, we attempted to predict functions of *SOX9-AS1* by means of a correlation analysis based on RNA-seq data between the expression levels of *SOX9-AS1* and genes in TNBC and basal-like samples from TCGA. The expression of *SOX9-AS1* in TNBC samples was positively correlated with 5944 and negatively correlated with 723 genes (Spearman rank, p value < 0.05). While in basal-like samples, the expression of *SOX9-AS1* was positively correlated with 4,503 and negatively correlated with 711 genes (Spearman rank, p value < 0.05) (Supplementary Table 8). The top 5 genes (positive- and negative-correlation) in TNBC and basal-like are shown in Fig. 4A,B, respectively. In TNBC, a strong positive correlation was observed between *SOX9-AS1* and *MIA* (r = 0.63, p value ≤ 0.0001) and *MIA-RAB4B* (r = 0.65, p value ≤ 0.0001) (Fig. 4C). Furthermore, these genes showed significantly higher expression levels in the basal-like molecular subtype (Fig. 4E). In contrast, a moderate positive correlation with the *ST3GAL6* (r = 0.49, p value ≤ 0.0001) and *CDH19* (r = 0.47, p value ≤ 0.0001) genes was observed in basal-like BRCA (Fig. 4D).

We then reasoned that *SOX9-AS1* may exert a survival effect on patients through its related genes, i.e., the highly correlated genes *MIA* and *MIA-RAB4B*. Thus, we performed a Kaplan–Meier analysis in order to assess this hypothesis. As shown in Fig. 4F, high mean expression of *SOX9-AS1*, *MIA* and *MIA-RAB4B* is significantly associated with an increased OS rate in basal-like BRCA patients (HR = 0.41; CI [0.22–0.79], p value = 0.006). We then performed the same analysis including the moderately correlated genes *ST3GAL6* and *CDH19*, which did not yield statistically significant results. Altogether, these data suggest that *SOX9-AS1*, *MIA* and *MIA-RAB4B* expression are not just statistically correlated, but indeed biologically associated or even co-regulated in this model.

### SOX9-AS1 location is enriched in the cytoplasm of TNBC and Basal-like breast cancer cell lines

**Figure 5:**
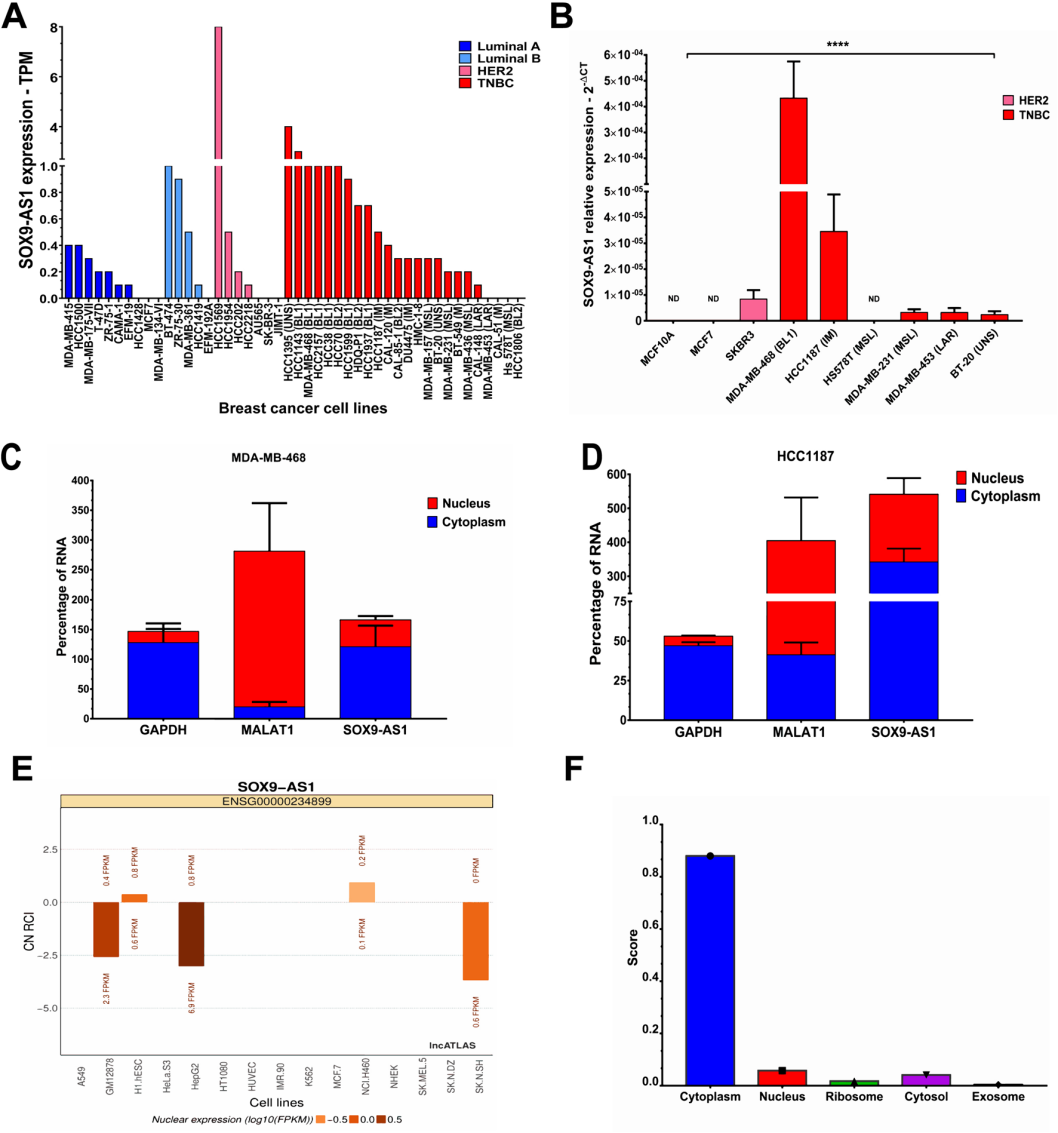
Expression and subcellular localization of *SOX9-AS1* in TNBC and basal-like cell lines. (**A**) Broad Institute Cancer Cell Line expression data of *SOX9-AS1* across 46 distinct BRCA cell lines examined by RNA-seq. (**B**) *SOX9-AS1* expression between BRCA cell lines examined by RT-qPCR. Mean ± SD. One-way ANOVA comparison test, ****p < 0.0001. (**C**) Subcellular localization of *SOX9-AS1* in MDA-MB-468, and (**D**) HCC1187 cells was determined by by RT-qPCR after nuclear/cytoplasmic fractionation using *MALAT1* as control for nucleus and *GAPDH* as control for cytoplasm. Analyses were performed three times in triplicate in vitro*.* (**E**) Subcellular localization of *SOX9-AS1* in various cell lines predicted using the lncATLAS database. (**F**) Subcellular localizations of *SOX9-AS1* determined by lncLocator predictor which includes RNA localization data from experimental evidence. ND: not detected.

To further understand the functional role of *SOX9-AS1* in TNBC cell lines, we examined its expression by RT-qPCR in various cell models of BRCA. Results showed that *SOX9-AS1* was enriched in cell lines classified as TNBC or basal-like in comparison to luminal and HER2 subtypes (Fig. 5A,B). Through subcellular fractionation, we show that *SOX9-AS1* is located mainly in the cytoplasm of MDA-MB-468 and HCC1187 cell lines, which correspond to the BL1 and IM subtypes of TNBC, respectively (Fig. 5C,D). We then predicted the subcellular localization of *SOX9-AS1* in five cell lines through the LncAtlas website (http://lncatlas.crg.eu/). *SOX9-AS1* was found to be distributed in the cytoplasm of both H1.Hesc (H1 human embryonic stem cell line) and NCI.H460 (non-small-cell lung cancer cell line), and in the nucleus of GM12878 (B-lymphoblastoid cell line), HepG2 (human hepatocellular carcinoma cell line), and SK.N.SH cells (human neuroblastoma cell line), but was not described in the MCF-7 luminal BRCA cell line (Fig. 5E), and LncLocator predictor showed that *SOX9-AS1* was localized to the cytoplasm (Fig. 5F). These results show that *SOX9-AS1* could have a biological role in cytoplasm.

### Knockdown of SOX9-AS1 potentially regulates genes involved in metabolic and immunological processes and pathways

**Figure 6:**
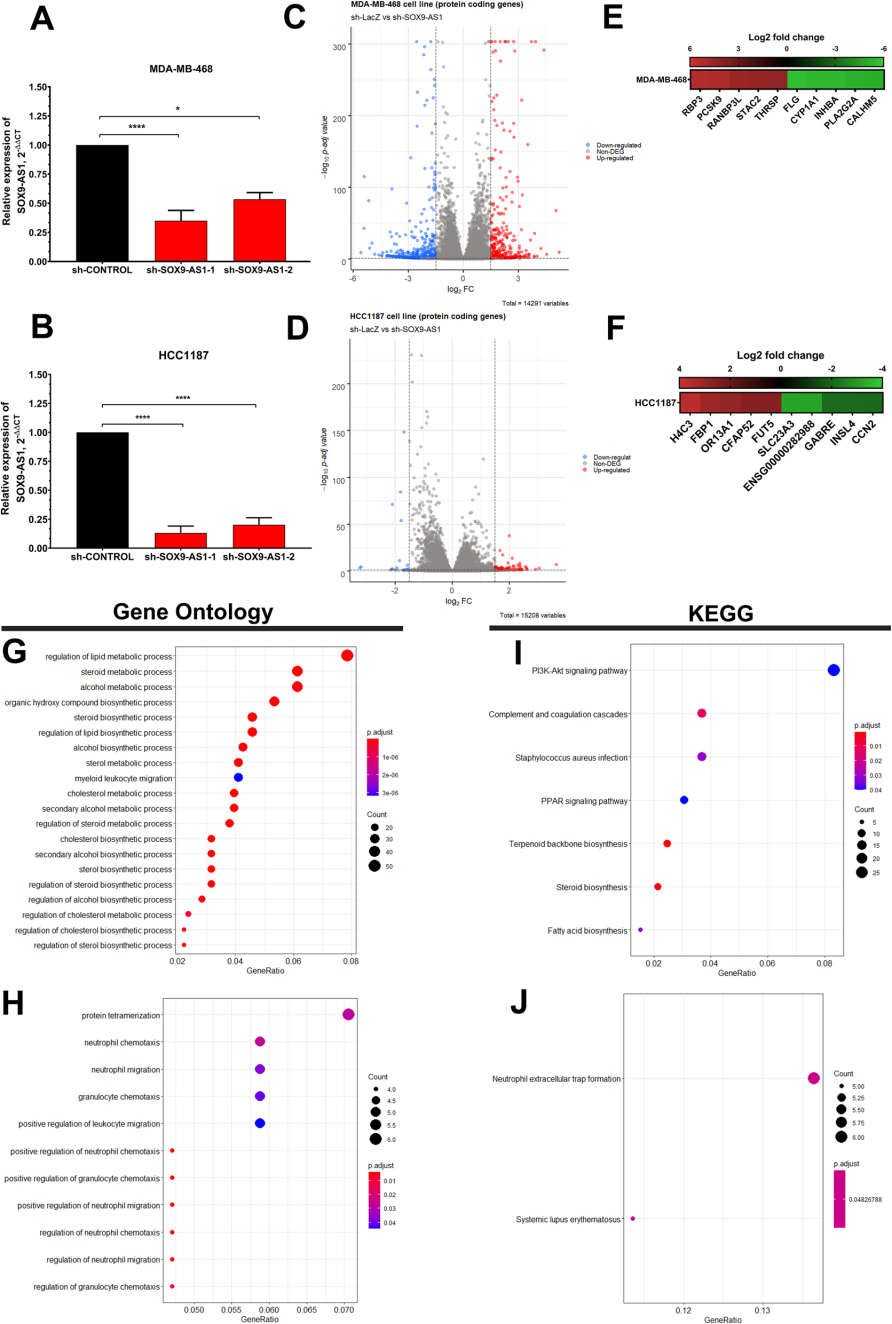
Knockdown of *SOX9-AS1* and DEGs involved in metabolic and immunological processes and pathways in TNBC cells in vitro. Analysis of *SOX9-AS1* levels by RT-qPCR assays in (**A**) MDA-MB-468, and (**B**) HCC1187 cells transfected with two *SOX9-AS1*-targeting shRNAs and control shRNA (sh-Control). Student T test. *p < 0.05, ****p < 0.0001. The Volcano plots represent the distribution of DEGs. Blue genes are downregulated, red genes are upregulated, and gray genes are not differentially expressed in (**C**) MDA-MB-468, and (**D**) HCC1187 cells. Up- and downregulated genes were defined as Log2 Fold Change ≤ − 1.5 and > 1.5 with adjusted p values < 0.05. Heatmap of the DGEs contributing to the top 5 in (**E**) MDA-MB-468 and (**F**) HCC1187 cells. Green: low expression, red: high expression according to Log2 fold change. Bubble map for the top 20 enriched GO classifications in (**G**) MDA-MB-468, and (**H**) HCC1187 cells. The most enriched KEGG pathways of DEGs in (**I**) MDA-MB-468, and (**J**) HCC1187 cell models. The size of the q-value is represented by the point’s color.

In order to elucidate the functional role of *SOX9-AS1* in basal-like and TNBC biology, we performed gene silencing experiments using two shRNAs (see “Materials and methods”). *SOX9-AS1* expression in *SOX9-AS1*-silenced cells was significantly reduced in MDA-MB-468 and HCC1187 (Fig. 6A,B). Moreover, after silencing, we assessed gene expression levels on a global scale using RNA-seq and evaluated genes that significantly changed their expression (Log2FC < − 1.5 to > 1.5, adjusted p-value < 0.05). In MDA-MB-468 cells, we found 740 DEGs. Of those, 44.7% (331 mRNAs) were upregulated, while 55.3% (409 mRNAs) were found to be downregulated when comparing the sh-control and the silenced SOX9-AS1-1 group (Fig. 6C). In the HCC1187 cell line, 100 coding genes were deregulated, of which 85 were upregulated and 15 were downregulated compared to control (Fig. 6D). 10 DEGs were shared between MDA-MB-468 and HCC1187 cells (Supplementary Table 9). Figure 6E,F show the top 5 DEGs in MDA-MB-468 and HCC1187, respectively. Additionally, we identified a total of 351 differentially expressed lncRNAs in MDA-MB-468 cells, of which 120 (34.2%) were upregulated and 231 (65.8%) downregulated when *SOX9-AS1* expression was inhibited (Supplementary Fig. 3A,B). In the HCC1187 cell line, we identified 56 differentially expressed lncRNAs, 32 were upregulated and 24 were downregulated (Supplementary Fig. 3C,D; Supplementary Table 10).

Interestingly, according to GO analysis, genes enriched participate in lipid, steroid, alcohol and cholesterol metabolism, biosynthetic processes, and a myeloid leukocyte migration process in MDA-MB-468 cells (Fig. 6G). Leukocyte, granulocyte, neutrophil chemotaxis and migration processes were mainly enriched in HCC1187 under the effect of *SOX9-AS1* silencing (Fig. 6H) (Supplementary Table 11). KEGG analysis ranked by enrichment score and p value < 0.05 in the MDA-MB-468 cell line showed pathways related to cancer and lipid metabolism, such as PI3K-Akt signaling and PPAR signaling, as well as steroid and fatty acid production (Fig. 6I). These findings are consistent with the biological processes and pathways identified in the DGE analysis in TNBC and basal-like patient samples (Fig. 2). Only two pathways were enriched in the HCC1187 cell line (Fig. 6J) (Supplementary Table 12). These results indicate a major biological role for *SOX9-AS1* as a potential regulator of genes that participate in cancer metabolism-related processes and inflammatory pathways in TNBC and basal-like subtypes.

### SOX9-AS1 regulates genes involved in metabolic processes and pathways in TNBC and Basal-like cells

**Figure 7:**
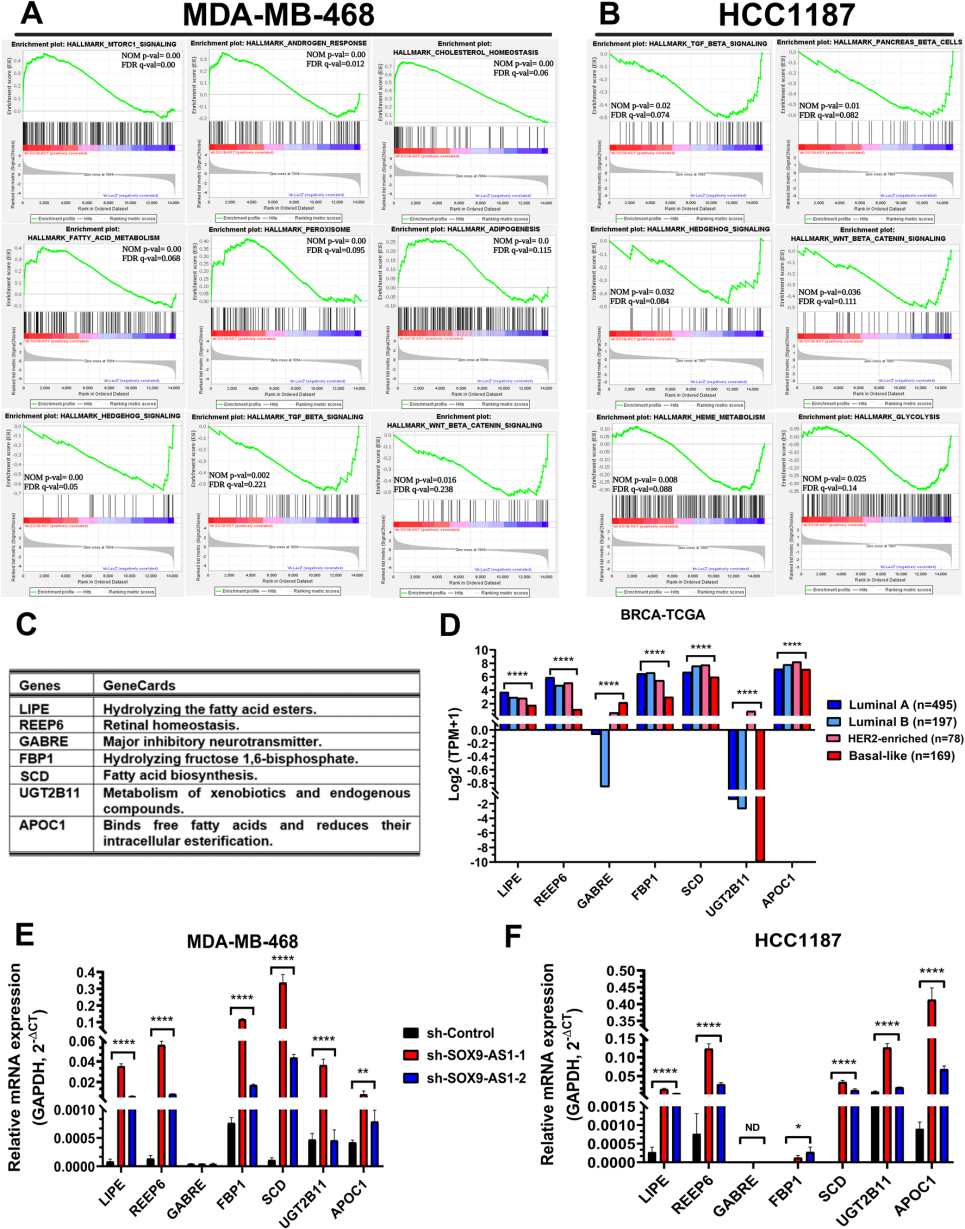
*SOX9-AS1* potentially regulates a variety of signalling pathways and metabolic cellular processes in TNBC cells. GSEA for the hallmark gene sets after *SOX9-AS1* silencing showed significant positive and negative enrichment (FDR q-value ≤ 0.25) in (**A**) MDA-MB-468, and (**B**) HCC1187. The NOM p-val and FDR q-value are displayed. (**C**) Seven genes involved in cellular metabolism were validated. (**D**) Distribution of candidate genes across PAM50-subtypes from BRCA-TCGA cohort. Data represent median expression value. Dunn's multiple comparisons and Kruskal–Wallis tests (****p < 0.0001). Validation of gene expression by RT-qPCR in (**E**) MDA-MB-468, and (**F**) HCC1187 cells transfected with two *SOX9-AS1*-targeting shRNA and sh-Control. Bar graph shows the quantified results with means ± SD of three biological replicates. Tukey’s test (*p < 0.05 and ****p < 0.0001 vs. sh-Control). ND: not detected.

We further performed GSEA analysis using the Hallmarks gene set. Considering nominal p value < 0.05 and FDR q value < 0.25. The most representative gene sets positively correlated with the silencing of *SOX9-AS1* in the MDA-MB-468 cell line were: mTORC1 signaling, androgen response, cholesterol homeostasis, fatty acid metabolism, peroxisome signaling, and adipogenesis, while the most representative negatively correlated gene sets were Hedgehog signaling, TGF-beta signaling, WNT/beta catenin signaling (Fig. 7A), and several other cellular processes (Supplementary Table 13). GSEA analysis in the HCC1187 cell line showed that the silencing of *SOX9-AS1* was only negatively correlated with hallmark gene sets, such as TGF-β signaling, pancreatic beta cells, Hedgehog signaling, estrogen response early, heme metabolism, and glycolysis (Fig. 7B). Furthermore, diverse mechanisms were fairly similar to those discovered in the MDA-MB-468 cell line (Supplementary Table 13). These results suggest that the biological role of *SOX9-AS1* is directly related to metabolic processes and pathways in both TNBC and basal-like subtypes.

We next validated some DEGs poorly studied in TNBC and basal-like cells, but involved in cellular metabolism (Fig. 7C). First, we used the BRCA-TCGA cohort to determined the expression of these genes in different BRCA subtypes (Fig. 7D) and observed that the expression level of *LIPE*, *REEP6*, *FBP1*, *SCD1*, *UGT2B11* and *APOC1*, were significantly underexpressed (p value = 0.0001) while *GABRE* expression was significantly increased in basal-like tumors (p value = 0001) compared to receptor-positive tissues (Fig. 7D). Then, we used RT-qPCR in our *SOX9-*AS1-silenced cell lines and found an opposite effect on the relative quantification of these seven genes when compared to the sh-Control in both MDA-MB-468 and HCC1187 cell lines (Fig. 7E,F). Thus, we confirmed that these genes were potentially regulated by *SOX9-AS1* in TNBC and basal-like subtypes.

### Knockdown of SOX9-AS1 stimulates fatty acid synthesis and intake in TNBC and Basal-like cells

**Figure 8:**
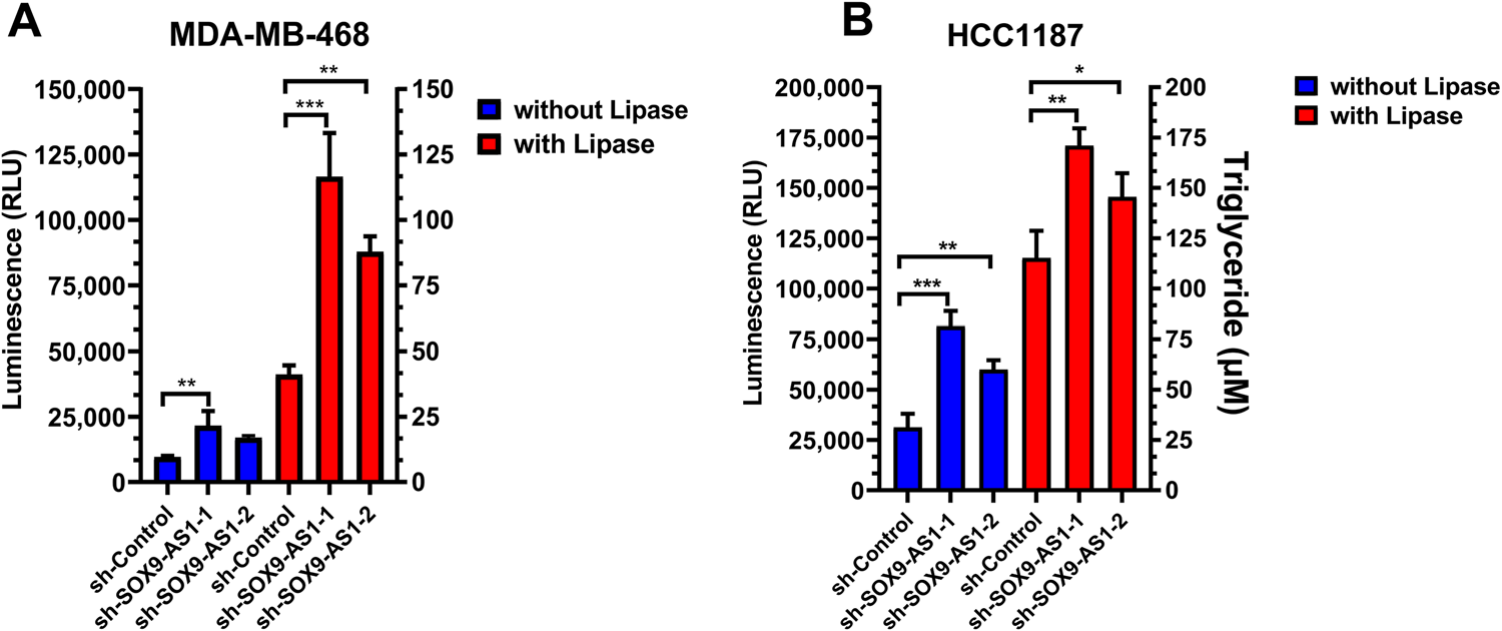
*SOX9-AS1*-knockdown increases intracellular triglyceride levels in TNBC cells. Intracellular triglycerides were quantified as glycerol (in relative light units-RLU) following treatment with or without Lipase in (**A**) MDA-MB-468 and (**B**) HCC1187 cells transfected with sh-control, sh-SOX9-AS1-1 or sh-SOX9-AS1-2. Values represent mean ± SD for three biological replicates. One-way ANOVA analysis for each cell line. *p < 0.05, **p < 0.001, ***p < 000.1.

Pathway analysis showed that many DEGs were significantly enriched in lipid metabolism, both in samples and cell lines (Figs. 3, 6, 7). Therefore, we explored whether *SOX9-AS1* participates in triglyceride metabolism (lipid compounds of fatty acids and glycerol) in TNBC cells. The levels of triglycerides in both MDA-MB-468 and HCC1187 cells after *SOX9-AS1* knockdown were significantly increased compared with those in control cells (p value < 0.01) (Fig. 8A,B). Furthermore, we used lipoprotein lipase to convert triglycerides into glycerol, since triglycerides are highly insoluble and difficult to titrate in aqueous solution^28^. We found that there was a greater amount of glycerol in *SOX9-AS1*-silenced TNBC cells *versus* control cells (Fig. 8A,B). Additionally, we evaluated the capacity of *SOX9-AS1* deficient-cells to accumulate intracellular lipids using the Oil Red O stain. However, the neutral lipids were not observed in *SOX9-AS1*-knockdown cells *versus* control cells (data not shown). These results showed that the loss of *SOX9-AS1* increases intracellular triglyceride levels in TNBC cells.

### Knockdown of SOX9-AS1 increases migration, and invasion of MDA-MB-468 and HCC1187 cells

**Figure 9:**
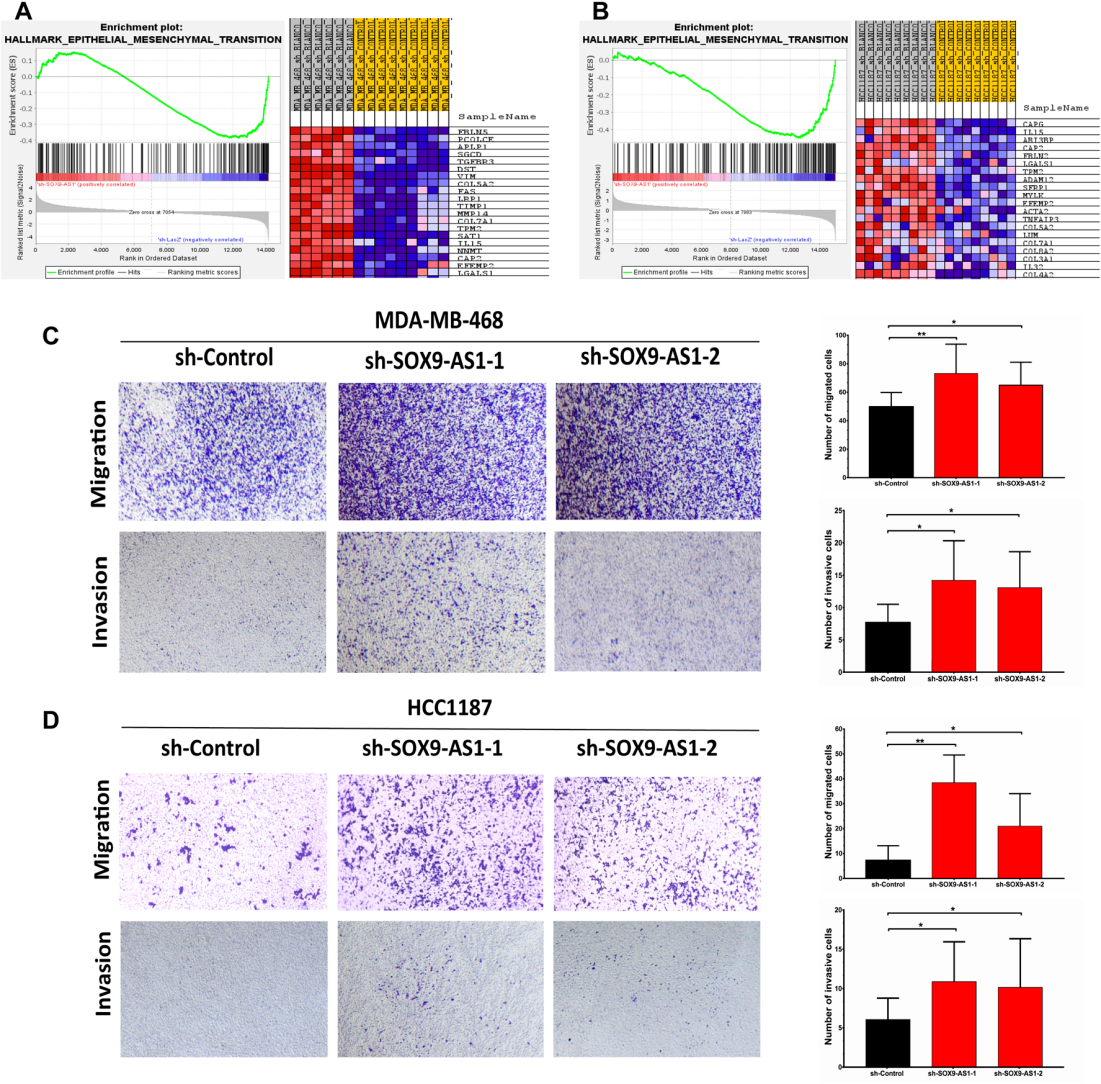
Knockdown of *SOX9-AS1* increases cell migration and invasion in TNBC cells. The DEGs obtained by RNA-seq data from the *SOX9-AS1*-knockdown in (**A**) MDA-MB-468 and (**B**) HCC1187 cells was analyzed with GSEA enrichment plots for the HALLMARK_EPITHELIAL_MESENCHYMAL_TRANSITION gene set. (**C**) Representative images of transwell migration and invasion assays after *SOX9-AS1*-knockdown in MDA-MB-468 cells and (**D**) HCC1187 cells and corresponding control cells. Bar graph shows the quantification of the results with means ± standard deviation. *p < 0.05 and **p < 0.01 vs. sh-Control.

To further address and identify the molecular events induced by *SOX9-AS1* in TNCB and basal-like subtype, we carried out a GSEA analysis that showed that *SOX9-AS1* deregulated genes enriched in the process of epithelial-mesenchymal transition (EMT) in both MDA-MB-468 (NOM p value 0.04, FDR q value 0.14) and HCC1187 cells (NOM p value 0.02, FDR q value 0.08) (Fig. 9A,B). Given these observations, we performed migration and invasion assays. *SOX9-AS1* silencing significantly increases the migration and invasion of MDA-MB-468 cells (p value ≤ 0.05 and < 0.01 for sh-SOX9-AS1-1 and sh-SOX9-AS1-2, respectively) (Fig. 9C) and HCC1187 cells (p value ≤ 0.05 for both sh-SOX9-AS1) (Fig. 9D) in comparison to control cells. Finally, these results confirm that loss of *SOX9-AS1* expression results in a more aggressive phenotype for TNBC cells.

## Discussion

LncRNAs participate in the regulation of the Hallmarks of cancer, favoring or inhibiting oncogenesis^29,30^. In this context, we evaluated the clinical implication and the biological role of *SOX9-AS1* in basal-like and TNBC subtypes using the BRCA-TCGA cohort and a panel of BRCA cell lines. First, we evidenced the expression levels of *SOX9-AS1* across different cancer types compared to normal tissues. We found that there were higher expression levels of *SOX9-AS1* in normal tissues *versus* cancer tissues, mainly those with secretory function (i.e., BRCA, TGCT, SKCM, PAAD). Additionally, we showed that some tumor types, like those of the nervous system (i.e., LGG, GMB), connective tissues (CHOL) and other excretory type cancer tissues (i.e., KIRP, KIRC) have higher expression levels of *SOX9-AS1* compared to normal tissue. Additionally, tumors related to white blood cells presented null or low levels of expression of *SOX9-AS1* (Fig. 1A). Due to these findings, we suggest that *SOX9-AS1* is ubiquitously expressed but may have different tissue-specific functions. This has been shown to be the case for some lncRNAs that show different expression patterns between different types of normal and tumor tissues and therefore fulfill different functions^31^. An example is the *FAM83H-AS1* lncRNA which is significantly overexpressed in 15/33 different tumor types, and is downregulated in LAML, suggesting that different mechanisms might exist for non-solid tumors^32^. *LINC00460* was found to be overexpressed in five different types of cancer *versus* normal tissue (BRCA, COAD, HNSC, PAAD, and READ), while in two central nervous system cancers, *LINC00460* showed low expression, compared to normal tissue counterparts (GBM and LGG), and a lack of expression in LAML^33^. This indicates that the biological role of lncRNAs is precise and closely related to spatial expression patterns between tissue types, and their dysfunction in cancer often influences disease development and progression^34,35^. Specifically, using BRCA-TCGA data, we revealed that the expression levels were significantly higher in TNBC and basal-like samples. These data strengthen our previous results using microarrays that show high expression of *SOX9-AS1* in TNBC and basal-like samples^18,19^. Based on these results, we propose that *SOX9-AS1* is a biomarker for these BRCA subtypes.

In addition, we found that *SOX9-AS1* expression varies with age, being higher in patients under 50 years of age and lower expression in the group of women over 50 years (Supplementary Table 1). This may be due to the epidemiological finding that TNBC primarily affects young, premenopausal women between the ages of 40 and 46, who make up about 15–20% of all breast cancer patients^36,37^. Furthermore, we also observed a significant positive association of *SOX9-AS1* expression with the basal-like subtype in the PAM50 molecular classification, being much higher that in the receptor-positive BRCA subtypes.

We believe that the strengths of our study are focused on the similarity of the processes and pathways identified in both samples and cell lines using the results from the DGE analysis of the TNBC and basal-like subtypes, which show genes enriched in hallmarks related to lipid metabolic reprogramming. Interestingly, we observed that *SOX9-AS1* potentially regulates genes which are similar to metabolic reprogramming signatures described in TNBC by Gong et al.^38^ (see Supplementary Fig. 4; Supplementary Table 14). In particular, genes potentially regulated by *SOX9-AS1* are enriched in the metabolic-pathway-based subtype 1 cluster (MSP1) which, in turn, is associated to the lipogenic subtype with upregulated lipid metabolism and with better clinical prognosis^38^. Collectively, *SOX9-AS1*-mediated modulation of these metabolic reprogramming-related genes can conceivably explain the effect of this lncRNA on the increased survival of TNBC patients (Fig. 2), since we observe that high *SOX9-AS1* expression in patients displayed an increased OS, DSS, and DFS in TNBC and basal-like patients. In this context, we observe that the expression of *SOX9-AS1* has a positive correlation with *MIA* and *MIA-RAB4B* genes in basal-like samples and that the increased expression of all three genes together show a better prognosis for patients with this molecular subtype (Fig. 4). We cannot discard, however, that some of the *SOX9-AS1* mediated regulation of immune-related pathways identified in this study may play a role in TNBC and basal-like biology and clinical features. Further functional studies must address this issue.

Lipid metabolic reprogramming is an emerging hallmark of malignancy^39^, and any abnormal changes in fatty acid metabolism seem to provide advantages for the growth of cancer cells^40^. In this study, we found that *SOX9-AS1* negatively regulates lipid levels in TNBC cells, since silencing this lncRNA increases triglyceride accumulation. This effect is probably regulated by thousands of genes modulated by this lncRNA. In this sense, we validated seven lipid metabolic-related genes (*LIPE, REEP6, GABRE, FBP1, SCD1, UGT2B11* and *APOC1)*, which are poorly studied in basal-like and TNBC. Interestingly, these genes, as well as many other DEGs found in our study, participate in lipid biosynthesis and fatty acid degradation in cancer (*SCD1, SCD5, FASN, ACSL1, ACSL4, ACSS2, LPIN1*), lipolysis (*LIPE, PLIN5, PNPLA3*), transport and uptake (*CD36, RBP3*) and lipid storage/lipid droplets (*ACAT2*) (see Supplementary Table 9)^39,41–43^. Additionally, we demonstrate that the activation of these genes by *SOX9-AS1* silencing, increases intracellular triglyceride levels in MDA-MB-468 and HCC1187 cell lines compared to control cells (Fig. 8). However, we did not observe intracellular lipid droplets, presumably because of increased lipid utilization for cell growth, proliferation, migration, invasion and cell survival of cancer cells^44,45^. This could also explain the increased EMT of the TNBC cells.

A previous study reported that *SOX9-AS1* expression is upregulated in hepatocellular carcinoma (HCC), as is *SOX9*, which regulates *SOX9-AS1* expression. In turn, *SOX9* activates the Wnt/beta-catenin pathway to promote EMT. Additionally, *SOX9-AS1* maintains increased *SOX9* expression by inhibiting miR-5590-3p, thereby promoting HCC progression and is therefore associated to poor outcome in HCC patients^21^. Another study showed that *SOX9-AS1* was overexpressed in tissues and cell lines of intrahepatic cholangiocarcinoma (ICC) and its upregulation was related to shorter OS and recurrence-free survival. Additionally, *SOX9-AS1* promotes growth, migration and invasion of ICC cells^23^. In contrast, our study shows that overexpression of *SOX9-AS1* predicts an improved clinical course in TNBC and basal-like patients based on TCGA and GEO databases. Furthermore, using the DGE data from TCGA, we identified that *SOX9-AS1* expression positively correlates with genes involved in the metabolic reprogramming of oxidative phosphorylation (OXPHOS), reactive oxygen species (ROS), and adipogenesis pathways in TNBC samples (Fig. 2), which are an important part of the metabolic phenotype of TNBC tumors^10,46^. Our experimental results show that *SOX9-AS1* is located mainly in the cytoplasm where it can fulfill its functional role. Based on our RNA-seq data of SOX9-AS1-knockdown TNBC cells, this lncRNA positively correlates with a plethora of genes involved in metabolic reprogramming of glycolysis, fatty acid metabolism, peroxisome pathway, and adipogenesis (Fig. 6), a major metabolic program that could provide a survival advantage to TNBC cells^10^. Moreover, these processes are triggered by mTOR signaling and PI3K-Akt-mTOR signaling (Fig. 6), which are responsible for driving tumorigenesis and modulating metabolism in cancer cells^47^. Additionally, silenced *SOX9-AS1* negatively correlates with genes involved in Hedgehog, TGF-β, and WNT signaling pathways (Fig. 7). These pathways trigger intracellular kinase cascades to induce transcription factors that activate the expression of EMT-associated genes^48^. In this sense, the in vitro results of the present study also demonstrate that *SOX9-AS1* can negatively regulate EMT of MDA-MB-468 and HCC1187 cells, since cell migration and invasion were increased in *SOX9-AS1*-knockdown TNBC cells. Studies reveal that cancer-associated EMT requires complex and comprehensive metabolic reprogramming, including gene expression and epigenetic changes^49^. Therefore, transcription program switching in EMT is induced by different signaling pathways, such as transforming growth factor b (TGF-b) and bone morphogenetic protein (BMP), Wnt-β-catenin, Notch, Hedgehog, and receptor tyrosine kinases^48^. Although the number of studies involving *SOX9-AS1* is limited and has more frequently been described as a predictive factor for poor prognosis, as shown for HCC, and ICC cells, we suggest that high expression of *SOX9-AS1* might serve as a biomarker of good prognosis in TNCB tissue specific biological mechanisms in the cytoplasm of TNBC cells. Taken together, our results suggest that the loss of expression of *SOX9-AS1* in TNBC cells promotes a more aggressive phenotype.

## Conclusions

Our study demonstrates that *SOX9-AS1* is a master regulator of a plethora of genes involved in metabolic reprogramming, cell migration and invasion in patient samples and cell models, and its biological function is influenced by the dynamic properties of EMT through different signaling pathways. Our results further our understanding of the important role of *SOX9-AS1* in the molecular cell biology of TNBC and basal-like subtypes. In this context, we suggest that *SOX9-AS1* could act as an onco-suppressor, since its elevated expression levels can result in a better clinical outcome for basal-like and TNBC.

## Materials and methods

### Analysis of SOX9-AS1 in the GEPIA2 (Gene Expression Profiling Interactive Analysis) database

*SOX9-AS1* expression levels were screened in 31 TCGA adjacent tissue and tumor datasets and GTEx normal tissues (unrelated donors) using the GEPIA2 database (http://gepia2.cancer-pku.cn/#index)^50^. For our research, we screened 291 normal tissues, of which 179 were healthy normal from GTEx data and 112 adjacent tumor tissues from the TCGA-BRCA cohort, and 810 BRCA samples. The 31 tumors included in the analysis are enlisted in Supplementary Materials. Bar plots were generated to visualize the median expression levels transformed to Log_2_(TPM+1).

### BRCA-TCGA dataset mining and stratification according to SOX9-AS1 expression

Clinical information from TNBC and its molecular classification by PAM50 were obtained from the TCGA database through cBioPortal (https://www.cbioportal.org/), and raw RNA-seq expression data was obtained from the GDC Data Portal (https://portal.gdc.cancer.gov/projects). Female patients who received neoadjuvant treatment or that lacked overall survival (OS), disease-specific survival (DSS), disease-free survival (DFS) and progression-free survival (PFS) data were excluded from this analysis. According to these criteria, 112 TNBC and 167 basal-like samples were included in this study. The expression data was normalized to Log_2_(TPM+1) and was used to stratify patients into groups with low and high expression of *SOX9-AS1* (NCBI Reference Sequence: NR_103738.1), (Ensemble ID: *ENSG00000234899.11*), based on the lower (25%) and upper quartiles (75%), respectively.

### Survival analyses

Kaplan–Meier survival analyses for OS, DSS, DFS and PFS were performed with the Log-rank test for BRCA-TCGA cohorts. p-values < 0.05 were considered statistically significant. Groups of patients with low and high expression of *SOX9-AS1* were stratified based on the lower (25%) and upper quartiles (75%), respectively. The analyses were performed with survival (v. 3.1-8) and survminer (v. 0.4.8) R packages. To analyze OS and RFS of GEO-derived cohorts we used the Kaplan Meier Plotter online tool (https://kmplot.com/analysis/), using the automatic cutoff calculation and JetSet probes only^51^. The Breast RNA-seq Kaplan Meier Plotter tool was used for the three-gene (*SOX9-AS1, MIA* and *MIA-RAB4B*) combined survival analysis in basal-like patients, using the mean expression of the genes, and the auto-detection cutoff parameter.

### SOX9-AS1-genes correlation analysis

To determine relationships between the *SOX9-AS1* expression and gene expression, we performed Spearman correlation analysis. p-value < 0.05 was considered statistically significant. This analysis was performed in the R platform (v. 4.2.2).

### Cell culture

The BRCA cell lines (MCF7, ZR-75-1, MDA-MB-361, SKBR3, MDA-MB-468, HCC1187, HS578T, MDA-MB-231, MDA-MB-453, BT-20) and non-tumorigenic epithelial cell line MCF10A, were purchased from American Type Culture Collection (ATCC, Manassas, VA, USA). Cell lines were routinely maintained in RPMI-1640 or DMEM medium (ATCC), supplemented with 10% fetal bovine serum (FBS) (ATCC), in a humidified incubator at 37 °C with 5% CO_2_ using the standard media and conditions established by the ATCC, except for MDA-MB-468 cell line, which was maintained in DMEM/High Glucose (Gibco, Grand Island, NY) with 10% FBS (ATCC).

### RNA extraction and reverse transcription quantitative PCR (RT-qPCR) assay

Total RNA was extracted from SOX9-AS1-deficient BRCA cell lines using TRIzol^®^ reagent (Invitrogen; Thermo Fisher Scientific, Inc.), according to the manufacturer's protocol. Briefly, cDNA was generated with 100 ng of total RNA in a final reaction volume of 20 µl using the High-Capacity cDNA Reverse Transcription Kit (Applied Biosystems™, Foster City, California, USA) following the manufacturer’s instructions. The expression level of *SOX9-AS1* was detected using the TaqMan Noncoding RNA assays (Hs01107818_m1) and the following genes were validated using Taqman assays (Applied Biosystems): *LIPE* (Hs00943410_m1), *REEP6* (Hs00922109_m1*)*, *GABRE* (Hs00608332_m1), *FBP1* (Hs00983323_m1), *SCD* (Hs01682761_m1), *UGT2B11* (Hs01894900_gH), *APCO1* (Hs001555790_m1) using qPCR analysis (Applied Biosystems, Foster City, USA) in a QuantStudio™ 5 Real-Time PCR System (Life Technologies). The qPCR reaction contained 1 µl of cDNA, 5 µl 2× TaqMan Universal Master Mix (Applied Biosystems, ThermoFisher™ Scientific, Waltham, Massachusetts, USA), 0.5 µl TaqMan probe and 3.5 µl of nuclease-free water. The thermocycling conditions were as follows: initial denaturation at 95 °C for 5 min, 40 denaturation cycles at 95 °C for 30 s, hybridization at 50 °C for 30 s, and extension at 72 °C for 30 s. The mean cycle threshold (Ct) value for *GAPDH* (Hs99999905) housekeeping gene was used to normalize the raw Ct data and calculate 2^−ΔCt^. The 2^−ΔΔCt^ method was used to estimate the relative expression level of genes under treatment conditions^52^.

### Subcellular fractionation of TNBC and basal-like cell lines

Cellular fractionation was performed using PARIS™ Kit (Invitrogen Inc., Carlsbad, CA, USA), according to the manufacturer’s instructions. RNA was isolated from the nucleus and cytoplasm compartments in MDA-MB-468 and HCC1187 cells. Subsequently, the *SOX9-AS1* intracellular localization analysis, by RT-qPCR assay, was performed as described above. The data was normalized using the formula % of input = 100 × [2^(Ct total RNA − Ct RNA fraction). MALAT1 and GAPDH expression was used as nuclear and cytoplasmic controls, respectively.

### Knockdown of lncRNAS SOX9-AS1 and cell transfection of shRNAs

The online tool BLOCK-iT™ RNAi Designer (Thermo Fisher Scientific) was used for the design of the short hairpin RNAs (shRNAs) targeting *SOX9-AS1* and the LacZ control (sh-Control) was supplied in the kit. Post-transcriptional gene silencing of the canonical transcript (NR_103738.1) and one transcript variant (NR_103737.1) was performed with two shRNAs (sh-SOX9-AS1-1 and sh-SOX9-AS1-2). The sequences are shown in Supplementary Table 15. Briefly, the construction with two dsOligos capable of generating shRNAs in cells was carried out using the BLOCK-iT U6 RNAi Entry Vector Kit (Invitrogen, Carlsbad, CA, EE. UU.), following the manufacturer's instructions. Once the plasmids were generated, the transformation was carried out in the competent cells *Escherichia coli* One Shot TOP10, which were subsequently seeded in LB medium plates with 50 mg/ml kanamycin. After transformation, plasmids were isolated using the PureLink Hipure Plasmid Maxiprep kit (Invitrogen), according to the manufacturer's guidelines. To confirm the presence and correct orientations of dsOligo inserts into the plasmids, Sanger sequencing was performed. Plasmid transfection for the specific silencing of *SOX9-AS1* was carried out in the HCC1187 and MDA-MB-468 cell lines using Xfect Transfection Reagent (Clontech, California, EE. UU.). Five µg of plasmid were used, and the silencing efficiency was determined at 48 h post-transfection by measuring *SOX9-AS1* expression levels through RT-qPCR.

### RNA-sequencing libraries preparation and data processing

We performed RNA-seq on 12 samples, three independent replicas for each silencing condition (sh-Control and sh-SOX9-AS1-1 for MDA-MB-468 and HCC1187 cells). Paired-end RNA-seq libraries with fragments of 76 bp were generated from total RNA with the TruSeq^®^ Stranded mRNA Library Prep Kit (Illumina, CA, USA), following the manufacturer’s recommendations. Briefly, the protocol started with 1 μg of total RNA, which was purified and fragmented using poly-T oligo. Then, cDNA was synthesized, and adapter ligation was performed. The constructed libraries were amplified through PCR, and then were quantified with the Agilent 4200 TapeStation System using High Sensitivity D1000 ScreenTape (Agilent, CA, USA), according to the manufacturer's protocol. The sequencing of RNA-seq libraries was carried out on the NextSeq 500 platform (Illumina, USA) at an average depth of 23 million raw paired reads per sample. The general statistics are shown in Supplementary Table 15. Quality control of raw fastq files was determined using FastQC (v. 0.11.9) and MultiQC (v. 1.11). Adapter sequences, poor quality reads with Q values ≤ 22 and reads with fragment sizes ≤ 70 bp were filtered through Trimmomatic (v. 0.39). The alignment of raw reads to GRCh38 (*Homo sapiens*) reference genome and the generation of bam files were carried out with Hisat2 (v. 2.1.0) and samtools, respectively. Abundance estimation and mapped reads counting was performed with featureCounts (v. 2.0.0). All the analyses were carried out in the Unix-Bash environment of Linux platform.

### Differential gene expression analysis and functional annotation

DGE analysis was determined using DESeq2 (v.1.26.0). We used two groups of TNBC and basal-like TCGA data (*SOX9-AS1* low vs high expression) and cell lines (sh-LacZ vs sh-SOX9-AS1-1). Before DGE analysis with cell line data, we excluded a technical replica due to an unsuccessful silencing in the MDA-MB-468 cell line. Then, RNA-seq expression data with raw counts less than 10 were filtered. Up- and downregulated genes were defined as Log2FoldChange (LogFC) < − 1.5 and > 1.5 with p adjusted values < 0.05. Volcano plots and heatmaps were generated with EnhancedVolcano (v. 1.4.0) and Pheatmap (v. 1.0.12). Subsequently, the lists of differentially expressed genes (DEGs) were used to perform GO over-representation analysis for biological processes and KEGG pathway analysis using clusterProfiler (v. 3.14.3). For these analyses, we used minGSSize = 10 and maxGSSize = 500 as default parameters. The results were represented as dot plots and adjusted p values < 0.05 were considered statistically significant. All analyses were executed in R (version 3.6.2).

### Gene set enrichment analysis (GSEA)

Using the normalized RNA-seq expression data from groups of cell lines (sh-LacZ vs sh-SOX9-AS1-1) and BRCA-TCGA data (low vs high expression of *SOX9-AS1*) we performed GSEA, using the Hallmarks_all_v7.4 gene set (with min size = 10 and max size = 500 as default parameters). These analyses were performed with 1000 permutations. Gene sets with nominal p values < 0.05 and FDR < 0.25 were considered statistically significant^53^. The analyses were performed through GSEA software (version 4.1.0).

### Triglyceride measurement

Intracellular triglyceride levels in HCC1187 and MDA-MB-468 cell lysates were detected by luminescence using Triglyceride-Glo Assay (Cat. J3160, Promega, USA), according to the manufacturer’s instructions. Briefly, 10,000 transfected cells were cultured 24 h before the assay on a 96-well plate. After 24 h (48 h post-transfection), cells were washed twice with PBS. Then, 25 µl of glycerol lysis solution with and without lipase was added and cells were incubated at 37 °C for 30 min. Then, 25 µl of glycerol detection reagent containing reductase substrate and kinetic enhancer reagent was added to the lysates. After 1 h, the luminescence value was detected using a plate luminometer Synergy/HTX (BioTek instrument, Inc. Winooski, VT). The relative light unit (RLU) values were expressed as mean ± standard deviation. Experiments were performed in triplicate and repeated three times.

### Oil red O staining

For lipid staining in HCC1187 and MDA-MB-468 cells, 48 after transfection, cell monolayers were washed twice with PBS and fixed with 10% formalin (HT501128-4L Sigma-Aldrich, Sweden) for 1 h at room temperature. Cells were then stained with Oil Red O and incubated at room temperature for 15 min. Oil Red O solution was then removed and cells were washed four times with ddH_2_O. The cells were carefully visualized under the microscope.

### Cell migration and invasion assays

Cell migration and invasion assays were performed using a 24‐well transwell chamber with a pore size of 8 μm (Corning, USA) in the absence and presence of Matrigel (Corning) for migration and invasion assays, respectively, following to the manufacturer’s protocol. MDA-MB-468 and HCC1187 cells (5 × 10^4^ cells per well) were seeded in the upper of chamber. The medium containing 10% FBS was deposited in the lower chamber as a chemoattractant. 48 h post-transfection, the cells were fixed in 3.7% paraformaldehyde for 20 min and then stained with 0.5% crystal violet for 10 min. The cells in the upper chamber were removed with a cotton swab, and the images were captured under the microscope. The number of cells was counted by ImageJ software and the mean cell count of three independent membranes was defined as the migration or invasion index. Experiments were repeated three times.

### Statistical analysis

Experimental results were reported as mean ± standard deviation (SD) of three independent experiments in triplicate. A two-tailed paired Student's t-test was used to analyze the distribution between independent groups and 2 × 2 Chi-squared tests were used to identify associations between clinicopathological variables and *SOX9-AS1* expression levels, both statistical analyzes were performed using GraphPad Prism 8 software (GraphPad Software Inc., La Jolla, CA, USA). Multiple level Chi-squared tests (2xn), Bonferroni multiple correction and *post-hoc* analyses were performed using custom made functions (https://github.com/neuhofmo/chisq_test_wrapper) based on SciPy, Statsmodels and Itertools Python modules, using the pandas library^54^. The complete code is available at: https://github.com/mriosromero/Chi-square-tests-Cisneros-et-al-2023. p values < 0.05 were considered statistically significant.

### Supplementary Information

Supplementary Figures.

Supplementary Information.

Supplementary Table 1.

Supplementary Table 2.

Supplementary Table 3.

Supplementary Table 4.

Supplementary Table 5.

Supplementary Table 6.

Supplementary Table 7.

Supplementary Table 8.

Supplementary Table 9.

Supplementary Table 10.

Supplementary Table 11.

Supplementary Table 12.

Supplementary Table 13.

Supplementary Table 14.

Supplementary Table 15.

## Data Availability

The data presented in this study are not publicly available due to privacy. However, the data can be requested from the corresponding authors.. To review BioProject-NCBI accession PRJNA964471. Go to (https://www.ncbi.nlm.nih.gov/bioproject/PRJNA964471). Link from BioSample-NCBI (https://www.ncbi.nlm.nih.gov/biosample?Db=biosample&DbFrom=bioproject&Cmd=Link&LinkName=bioproject_biosample&LinkReadableName=BioSample&ordinalpos=1&IdsFromResult=964471).
