# Supplementary material for: LncRNA SOX9-AS1 triggers a transcriptional program involved in lipid metabolic reprogramming, cell migration and invasion in triple-negative breast cancer
Source: Sci Rep. 2024 Jan 17;14:1483. doi: 10.1038/s41598-024-51947-2 (PMC10794186; doi:10.1038/s41598-024-51947-2)

LncRNA *SOX9-AS1* triggers a transcriptional program involved in lipid metabolic reprogramming, cell migration and invasion in triple-negative breast cancer

Mireya Cisneros-Villanueva[1,2,3], Marco Antonio Fonseca-Montaño[1,4], Magdalena Ríos-Romero[1], César López-Camarillo[5], Silvia Jiménez-Morales[1], Elizabeth Langley[6], Alan Sajid Rosette-Rueda[1], Alberto Cedro-Tanda[7], Daniel Hernández-Sotelo[3]*, Alfredo Hidalgo-Miranda[1,*]

Supplementary figures

Supplementary figure 1. Differentially expressed lncRNAs in TNBC and basal-like samples. A) Venn diagram indicating the number of significant DGEs across two comparisons (TNBC and basal-like) and the overlap between each set of genes. The Volcano plot represents the distribution of differentially expressed lncRNAs. Blue genes are downregulated, red genes are upregulated, and gray genes are not differentially expressed in **(B)** TNBC-TCGA, and **(D)** basal-like-TCGA samples. Heat map showing the clustering of samples by lncRNAs that significantly increased (yellow font) or decreased (blue font) in **(C)** TNBC samples**,** and **(E)** basal-like samples of BRCA-TCGA cohort. Up- and downregulated genes were defined as Log2 Fold Change ≤ -1.5 and >1.5 with p adjusted values <0.05.

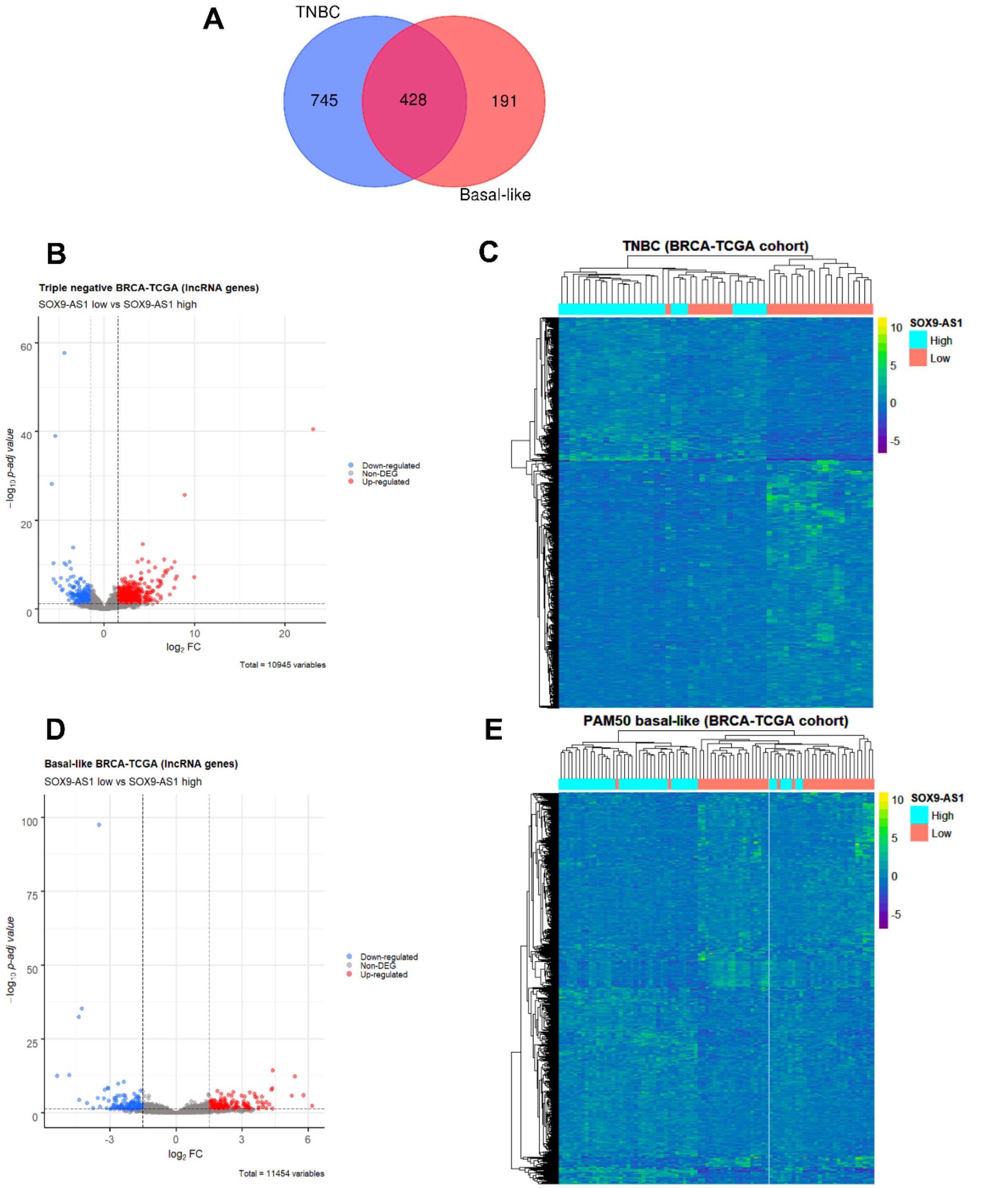

Supplementary figure 2. GO and KEGG pathway analyses of DEGs in TNBC and basal-like samples. Bubble map of the 20 most enriched GO classifications of DEGs and proteins between low and high expression in **(A)** TNBC-TCGA, and **(B)** basal-like-TCGA samples. The top 20 of KEGG pathways of DEGs in **(C)** TNBC-TCGA, and **(D)** basal-like-TCGA samples. The size of q-value is represented by the point´s color. The vertical axis represents the path name, and the horizontal axis represents the path factor corresponding to the Gene Ratio.

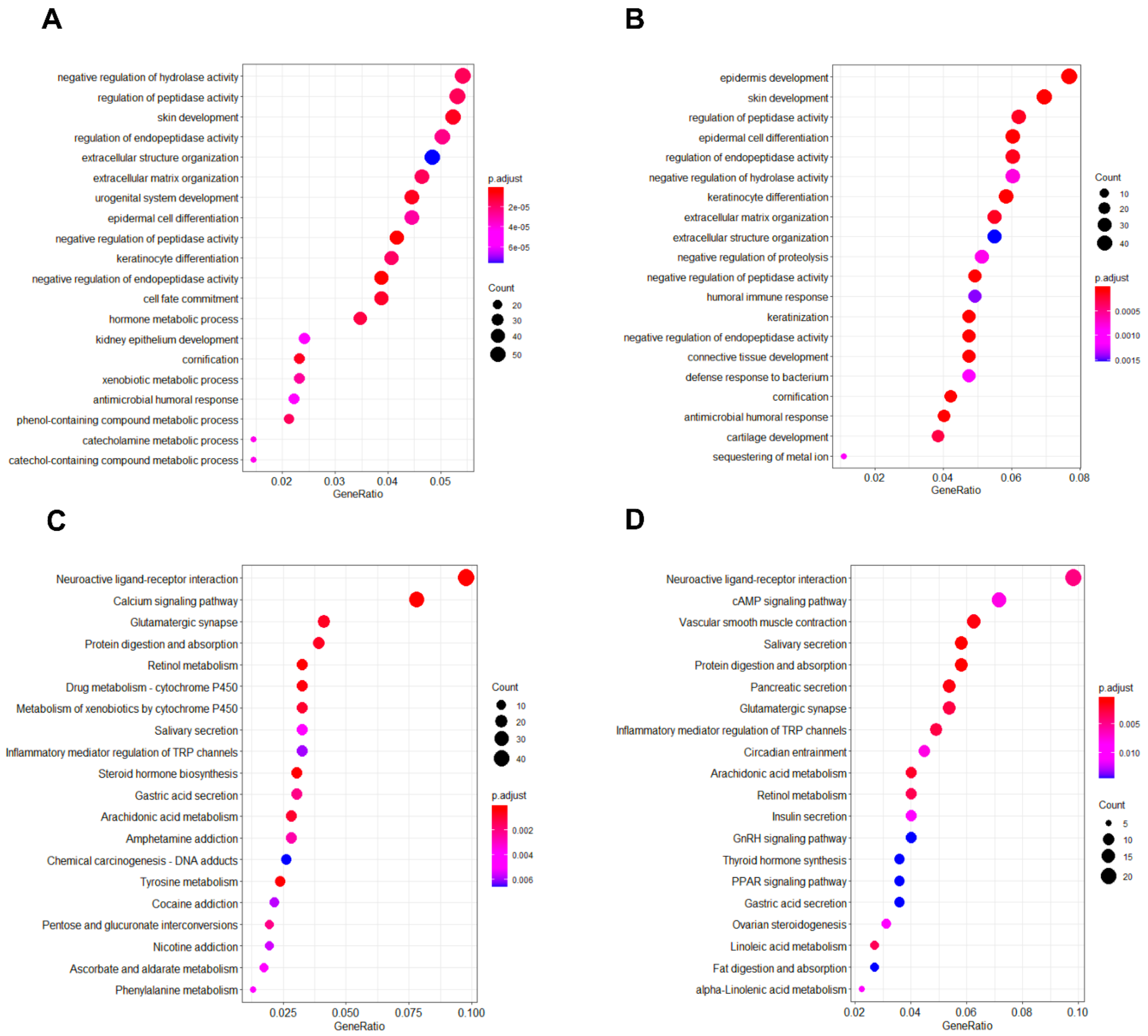

Supplementary figure 3. Differentially expressed lncRNAs in MDA-MB-468 and HCC1187 cells. The Volcano plot represents the distribution of differentially expressed lncRNAs. Blue genes are downregulated, red genes are upregulated, and gray genes are not differentially expressed in **(A)** MDA-MB-468 (molecular subtypes of TNBC—BL1), and (**C)** HCC1187 (molecular subtypes of TNBC—IM). Heat map showing the clustering of samples by lncRNAs that significantly increased (yellow font) or decreased (blue font) in **(B)** MDA-MB-468**,** and **(D)** HCC1187 cells. Up- and downregulated genes were defined as Log2 Fold Change ≤ -1.5 and >1.5 with p adjusted values <0.05.

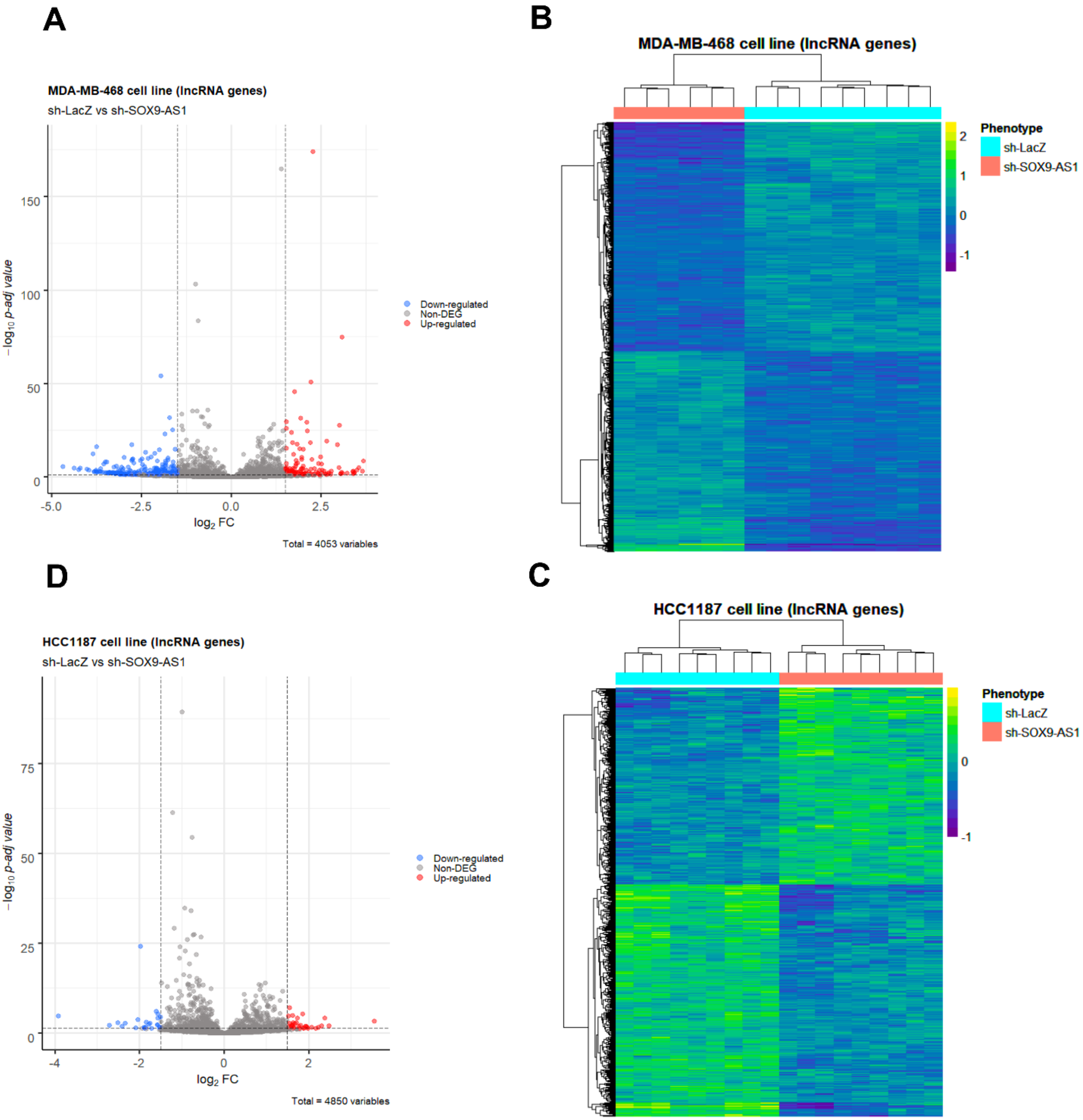

Supplementary figure 4. Number of *SOX9-AS1* regulated genes overlapping with metabolic-pathway-based subtypes (MPSs) found by Gong et al. (2021). Venn diagram indicating the number of significant DEGs across the two studies. MPS1, the lipogenic subtype with upregulated lipid metabolism; MPS2, the glycolytic subtype with upregulated carbohydrate and nucleotide metabolism; and MPS3, the mixed subtype with partial pathway dysregulation. SOX9-AS1, our study.

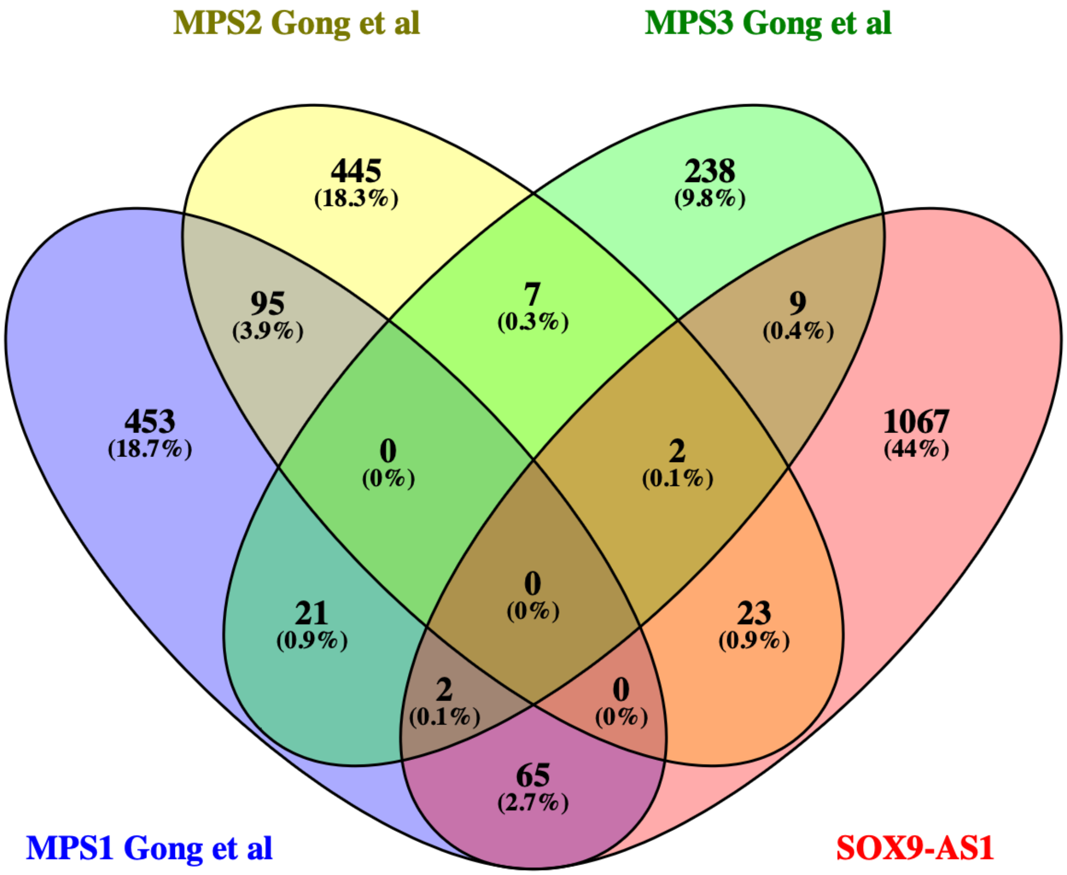

Supplement: Supplementary file 1 — Supplementary Figures. [file 41598_2024_51947_MOESM1_ESM.pdf]
