# Supplementary material for: LncRNA SOX9-AS1 triggers a transcriptional program involved in lipid metabolic reprogramming, cell migration and invasion in triple-negative breast cancer
Source: Sci Rep. 2024 Jan 17;14:1483. doi: 10.1038/s41598-024-51947-2 (PMC10794186; doi:10.1038/s41598-024-51947-2)
Supplement: Supplementary file 2 — Supplementary Information. [file 41598_2024_51947_MOESM2_ESM.docx]

LncRNA *SOX9-AS1* triggers a transcriptional program involved in lipid metabolic reprogramming, cell migration and invasion in triple-negative breast cancer

Mireya Cisneros-Villanueva^1,2,3^, Marco Antonio Fonseca-Montaño^1,4^, Magdalena Ríos-Romero^1^, César López-Camarillo^5^, Silvia Jiménez-Morales^1^, Elizabeth Langley^6^, Alan Sajid Rosette-Rueda^1^, Alberto Cedro-Tanda^7^, Daniel Hernández-Sotelo^3^*, Alfredo Hidalgo-Miranda^1,^*

**Supplementary Materials**

**TCGA Study Abbreviations:** Breast invasive carcinoma (BRCA); Testicular germ cell tumors (TGCT); Skin cutaneous melanoma (SKCM); Glioblastoma multiforme (GBM); Brain lower grade glioma (LGG); Pancreatic adenocarcinoma (PAAD); Sarcoma (SARC); Kidney renal papillary cell carcinoma (KIRP); Kidney renal clear cell carcinoma (KIRC); Kidney chromophobe (KICH); Thyroid carcinoma (THCA); Stomach adenocarcinoma (STAD); Cholangiocarcinoma (CHOL); Prostate adenocarcinoma (PRAD); Colon adenocarcinoma (COAD); Rectum adenocarcinoma (READ); Liver hepatocellular carcinoma (LIHC); Head and neck squamous cell carcinoma (HNSC); Lung adenocarcinoma (LUAD); Lung squamous cell carcinoma (LUSC); Esophageal carcinoma (ESCA); Pheochromocytoma and Paraganglioma (PCPG); Cervical squamous cell carcinoma and endocervical adenocarcinoma (CESC); Bladder urothelial carcinoma (BLCA); Adrenocortical carcinoma (ACC); Ovarian serous cystadenocarcinoma (OV); Uterine corpus endometrial carcinoma (UCEC); Uterine carcinosarcoma (UCS); Lymphoid neoplasm diffuse large B-cell lymphoma (DLBC); Acute myeloid leukemia (LAML); Thymoma (THYM).
